# Digital Therapeutics for People with Schizophrenia Spectrum Disorders: A Systematic Literature Review of Their Effect on Symptoms and Functioning

**DOI:** 10.1093/schbul/sbaf134

**Published:** 2025-08-25

**Authors:** Daniel Fulford, Christoph U Correll, Philip D Harvey, Alex S Cohen

**Affiliations:** Sargent College of Health & Rehabilitation Sciences, Boston University, Boston, MA 02215, United States; Department of Psychological & Brain Sciences, Boston University, Boston, MA 02215, United States; Department of Psychiatry and Molecular Medicine, The Donald and Barbara Zucker School of Medicine at Hofstra/Northwell, Hempstead, NY 11549, United States; Center for Psychiatric Neuroscience, Feinstein Institute for Medical Research, Manhasset, NY 11030, United States; Department of Child and Adolescent Psychiatry, Charité - Universitätsmedizin Berlin, 10117 Berlin, Germany; German Center for Mental Health (DZPG), partner site Berlin, 10117 Berlin, Germany; University of Miami Miller School of Medicine, Miami, FL 33136, United States; Department of Psychology, Louisiana State University, Baton Rouge, LA 70803, United States; Center for Computation and Technology, Louisiana State University, Baton Rouge, LA 70803, United States

**Keywords:** evidence-based software, smartphone app, engagement, efficacy, real-world functioning, quality of life

## Abstract

**Background and Hypothesis:**

Digital therapeutics (DTs; health software to treat/alleviate a disease/condition) may provide accessible evidence-based care to people with schizophrenia spectrum disorders (SSDs). This systematic literature review (SLR) investigated whether people with SSD find DTs acceptable and can use them effectively; whether DTs are effective and generally safe; and if DT use can impact functioning, quality of life (QoL), and other outcomes.

**Study Design:**

This PROSPERO-registered SLR (CRD42023476545) was conducted to identify articles assessing DTs in adults with SSD. Databases (MEDLINE/Embase/PsycINFO/Cochrane-SR/ClinicalTrials.gov/WHO International Clinical Trials Registry) and key congresses were searched up to January 28, 2024. Screening was completed by 2 reviewers, each blinded to the other’s decisions, and article summaries were extracted.

**Study Results:**

Of 886 unique citations, 115 eligible citations provided 38 unique primary studies with results and 10 linked citations. Engagement and satisfaction with DTs were generally high. Among 24 studies assessing the effectiveness of DT use on negative and positive symptoms, cognitive performance, mood, depression and/or anxiety, medication adherence, recovery, and general/other symptoms, 17 had ≥1 outcome showing significant change versus comparator/baseline; 24 had ≥1 outcome showing no significant difference. DTs were generally safe. Of 24 studies reporting real-world functioning, QoL, and other outcomes, 11 reported ≥1 significant outcome; 24 reported ≥1 outcome showing no difference versus comparator/baseline.

**Conclusions:**

People with SSD can use DTs effectively and find them acceptable. Although effectiveness outcomes varied across/within studies, DTs may provide an acceptable strategy for delivering accessible, generally safe, evidence-based care to people with SSD.

## Introduction

Schizophrenia is a complex psychiatric disorder characterized by positive symptoms (eg, delusions, hallucinations), negative symptoms (eg, social withdrawal, lack of motivation), disorganized speech and behavior, and deficits in cognitive performance (eg, working memory and processing speed), all of which can impair major areas of functioning and have a detrimental impact on quality of life (QoL).^1-3^ With the exception of cognitive remediation therapy, which targets cognition exclusively and was the subject of a recent extensive review,^4^ pharmacological or psychosocial treatment options for negative symptoms and cognitive deficits are lacking.^5^^,^^6^ There are also barriers that prevent people from engaging with evidence-based psychosocial therapies, including stigma, limited availability of resources, long wait lists, and lack of trained healthcare professionals to deliver therapy.^7-10^

One approach to helping people manage their symptoms has been the evolution of digital health. Digital health is a term applicable to the many technologies that engage patients in their health and wellbeing and encompasses mobile health (mHealth; a broad term covering evidence-based and non–evidence-based digital health), telemedicine (medical communication between different locations via electronic means, eg, videoconference), and wearable or smart devices (ie, internet-connected), as well as health information technology.^11^^,^^12^ Digital therapeutics (DTs) form a category of digital health and are defined as evidence-based software interventions used to help people manage their medical conditions.^11^^,^^13-15^ Common features of DTs designed for people with schizophrenia include daily activity reminders, action plans for goal setting, and prompts to help recognize early signs of psychosis.^16-18^ Other common features include ecological momentary interventions (EMIs). These may take a variety of forms, such as an unstructured clinical suggestion (eg, to practice a relaxation technique) or a more formal structured intervention that is tailored to the needs of an individual’s specific goal (eg, to reduce negative psychological symptoms).^19^ Ecological momentary interventions are often informed by responses to questionnaires and visual analog scales, which can be used to assess symptoms and track an individual’s progress.^17^^,^^20^

As many people with schizophrenia have access to smartphones,^21^ DTs alone or as an adjunct to other therapies have the potential to increase access to mental health services.^22^^,^^23^ They can provide support between face-to-face therapy sessions, facilitate continuity of care (eg, after discharge from inpatient treatment), and, theoretically, bridge the gap between referral and the beginning of in-person psychotherapy.^23^^,^^24^ Digital therapeutics that require prescription by a licensed clinician are known as prescription DTs (PDTs).^25^ In order to obtain approval for prescription, DTs require rigorous efficacy and safety testing by investigators and review by regulatory authorities.^26-29^ In the United States, the Food and Drug Administration (FDA) regulates DTs and PDTs that meet the definition for software as a medical device (SaMD), described as, “Software intended to be used for 1 or more medical purposes (eg, disease diagnoses, mitigation, treatment, or prevention) that perform these purposes without being part of a hardware medical device.”^30^ SaMD products are evaluated for potential risk to the device user and assigned to Class I (low), II (moderate), or III (high) accordingly; as PDTs specifically treat diseases, they are categorized as Class II devices.^31^ There are 2 FDA pathways to device authorization. One is the de novo pathway, in which clinical data demonstrating device safety and effectiveness are required and, once authorized, can serve as “predicates” for other devices.^32^ The other is the 510 k clearance pathway, which requires clinical data demonstrating substantial equivalence of safety and effectiveness to a predicate product previously authorized by the FDA.^33^^,^^34^ The FDA exercised enforcement discretion during the COVID pandemic by providing temporary or partial exemptions to the regulatory requirements for some PDTs due to the use of unique device identifier codes.^35^ Although several PDTs are under development for schizophrenia and some have received breakthrough device designation,^36^ none are currently approved in the United States by the FDA, in Europe by the European Medicines Agency, in Germany by the German Federal Institute for Drugs and Medical Devices (BfArM), or in the United Kingdom by the NHS’s Digital Health Technology Standard.

In recognition of this rapidly growing area of therapeutics and the need for better understanding of how best to implement and manage DTs among people with schizophrenia spectrum disorders (SSDs; ie, schizophrenia and schizoaffective disorder, diagnoses in our pre-defined search criteria), a systematic literature review (SLR) was conducted to explore the evidence base for DT use in this population based on 4 specific research questions (RQs): (1) Do people with schizophrenia/SSD use DTs and are they capable of using them effectively?; (2) what is the efficacy/effectiveness of DTs and PDTs in schizophrenia/SSD?; (3) what are the safety concerns associated with DTs and PDTs in schizophrenia/SSD?; and (4) what is the impact of DTs and PDTs in schizophrenia/SSD on real-world functioning, QoL, and other outcomes?

## Methods

An SLR was conducted to identify publications on studies of DT use in people with schizophrenia/SSD and is reported here per the Preferred Reporting Items for Systematic reviews and Meta-Analyses (PRISMA) guidelines.^37^ The protocol was registered with PROSPERO on November 11, 2023 (CRD42023476545), with updates (eg, to the title) made on March 18, 2025.^38^

### Search Strategy

MEDLINE, Embase, PsycINFO, and the Cochrane Database of Systematic Reviews were searched for relevant articles on November 13, 2023. The search strategy combined controlled vocabulary and keyword terms for the diagnosis (schizophrenia and schizoaffective disorder) and for DTs (including broad terms and names of known interventions in development); see Suppl. Table 1 for full search terms. No limits were placed on year or language of publication. The search was rerun on January 28, 2024, to identify any further relevant articles published in the intervening time. Clinical society websites were searched for abstracts (and posters, if available) published in the previous 2 years from the following annual conferences: American Psychological Association, Schizophrenia International Research Society, and the American Psychiatric Association. ClinicalTrials.gov and the WHO International Clinical Trials Registry Platform were searched for registered trials; publications linked to the ClinicalTrials.gov entry and results published as part of this entry were included (Suppl. Table 2). Bibliographies of systematic reviews identified during screening were reviewed to identify further relevant studies.

### Selection Process

Identified publications were screened at 2 levels (abstract and full text), based on predefined eligibility criteria (below). Reference screening software (DistillerSR) was used to manage deduplication of articles from multiple databases and dual-reviewer screening for study selection. Screening of each abstract and full text was completed by 2 reviewers (masked to each other’s decision) with reconciliation and a senior reviewer resolving any conflicts.

### Eligibility Criteria

Inclusion of articles was based on the criteria for eligibility, according to the Participant, Intervention, Comparison, Outcomes, Study design (PICOS) framework.

#### Participants

Eligible studies included adults with a diagnosis of schizophrenia, schizoaffective disorder, or SSD (without further specification of diagnosis). Studies including populations with a mix of diagnoses or in which individuals had experienced a first episode or early psychosis were included if it was established that ≥75% of the population were diagnosed with schizophrenia, schizoaffective disorder, or another SSD. The following studies were excluded: (1) those conducted exclusively in people <18 years of age; (2) those in which participants were receiving treatment in an inpatient setting; and (3) those in which the condition targeted by the intervention was not schizophrenia or schizoaffective disorder (eg, smoking or another substance use disorder), even if the study population included people with schizophrenia or schizoaffective disorder.

#### Intervention

Eligible studies included DTs (prescription or non-prescription) for the above populations. To be eligible, DTs needed to be delivered remotely (eg, via a smartphone app). Text messaging (SMS)–based interventions were included if they were interactive (eg, did not provide only passive reminders) and provided automated responses (eg, were not interactive telepsychiatry communications with service providers or peers). Interventions designed to only assess symptoms or interventions not designed specifically for people with schizophrenia or schizoaffective disorder (eg, transdiagnostic cognitive behavioral therapy [CBT]) were also excluded, as were interventions providing computerized training targeting neurocognition and social cognition. Further exclusions were studies of patient education websites, videos, social media platforms, and internet-based self-management tools that only provided self-management information, peer support, and healthcare professional support. Studies involving digital medicine systems (pill–device combinations for monitoring treatment adherence), digital devices with passive audio recording, or digital sensing for use in subsequent in-person therapy sessions were also excluded, as were studies speculatively exploring DTs without focusing on a specific intervention.

#### Comparison

Studies with any or no comparator were included.

#### Outcomes

Studies reporting the following outcomes for DTs were included: (1) adherence, acceptance, persistence, and any other measures of DT access and adherence in people with SSD; (2) efficacy or effectiveness in treating positive symptoms, negative symptoms, and/or cognition; (3) safety; and (4) measures of real-world functioning or QoL and other types of clinical, patient, or health system outcome measures reported with use of these interventions.

#### Study Design

Any type of interventional study, including controlled and uncontrolled studies, and any observational studies reporting outcomes for interventions of interest were included. Case reports were included to characterize the evidence base for interventions of interest, but outcome data were not extracted for this study type given its low evidence quality. Systematic literature reviews were identified during screening and used to identify any further studies of relevance. Given the number of SLRs identified during screening, non-systematic reviews, editorials, and commentaries on the topic were not included.

#### Data Synthesis

A summary of each identified study was extracted, including citation, name and description of DT, study aim, design, population, and outcomes. Additional extractions included a summary of key results, grouped by RQ, conclusions, and identified study limitations. Data were extracted by 1 reviewer. A second reviewer independently checked all data.

## Results

### Literature Search Results

**Figure 1:**
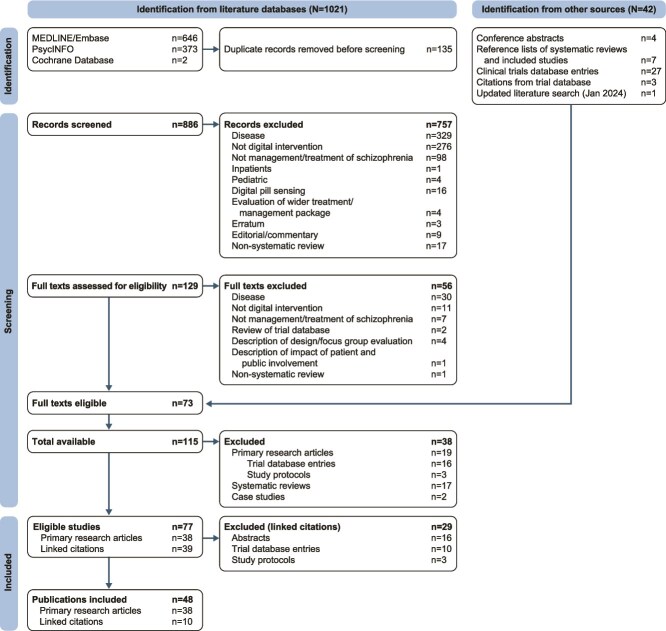
PRISMA flow chart.

Of 886 unique citations identified from literature databases, 757 were excluded based on abstract screening, and 56 were excluded following full-text review, leaving 73 eligible full texts (Figure 1). A further 42 eligible records were identified from other sources. Among 115 eligible articles, 38 were primary publications with results and 10 were linked citations with data from additional analyses (eg, secondary, exploratory, post hoc), totaling 48 citations covering 38 unique studies.

#### Study Characteristics

Of the 38 studies included, 17 were randomized controlled trials (RCTs), 1 was an interventional non-equivalent comparative study, and 20 were non-comparative studies. Study characteristics and descriptions of DTs are shown in Suppl. Table 3. Many studies related to more than 1 RQ; of the 38 studies, 35 were pertinent to RQ1, 24 to RQ2, 12 to RQ3, and 24 to RQ4. For RQ2 and 4, only studies assessing statistical differences (between treatment groups or within a treatment group over time) were considered in this review. Of note, while this review was designed to include articles on DTs and PDTs, this review focuses solely on DTs because no publications on approved PDTs were identified within the search period.

#### RQ1: Do people with schizophrenia/SSD use DTs, and are they capable of using them effectively?

Thirty-five of the 38 included studies contained data relating to RQ1; 16 were comparative and 19 were non-comparative studies (Suppl. Table 4). The use of DTs was assessed in a variety of ways across studies (eg, usability testing, adherence monitoring, and participant engagement assessments). Results also included participant satisfaction, allowing some insights into why DTs were/were not used as planned.

#### DT Use and Engagement

Engagement with DTs was generally high, as measured by the proportion of people using DTs during the studies (74%-97%) and/or the proportion of people responding to/sending text messages (67%-86%) or completing challenges/surveys (>64%).^17^^,^^20^^,^^24^^,^^39-58^ Notably, 2 studies found experience sampling method (ESM) completion rates to be higher in the evening than during the day.^20^^,^^44^ Moreover, in a study assessing the feasibility and validity of ExPRESS, a smartphone app used to report early signs, basic symptoms, and psychotic symptoms to predict relapse, percentage app use was significantly inversely correlated with baseline depression (*P* = .015) and fear of relapse (*P* = .014).^41^ Low levels of engagement were also seen in a small number of studies. For example, in a qualitative study of MindFrame, a smartphone app that allows young adults recently diagnosed with schizophrenia to access resources to aid their self-management, just 35% of individuals invited to use the app agreed to use it, with engagement waning over time.^16^ Additionally, in a non-comparative study combining in-person therapy with CBT2go, a smartphone app for mobile-assisted CBT for negative symptoms, just 22% and 19% of participants responded to action-plan prompts at weeks 12 and 24, respectively.^59^

Frequency and duration of app use were available in some reports. In a small, 1-month, non-comparative interventional trial of FOCUS, a smartphone app for automated real-time/real-place illness management support, trial completers used the app on 86.5% of the days they had the smartphone (average 5.9-6.7 days per week) and 5.2 times/day on average.^60^ For FOCUS-AV, average use was 6 days/week, 4 times/day.^39^ Frequency of use declined over time in several studies. For example, in a non-comparative study of an unbranded self-management app designed to support medication use and daily activities, daily recorded activities decreased from ~100 entries/day at study initiation to <20/day by the end of the month-long study.^18^ Similarly, in a non-comparative study of IMPACHS, a mobile solution based on CBT for psychosis (CBTp) covering monitoring, e-learning, and interventions into routine clinical care, participants used the app more frequently in month 1 (mean 4.25 days/week) than in month 5 (mean 2.10 days/week).^53^ Additionally, in a large, 3-6-month non-comparative study of FOCUS, engagement declined from 3.9 uses in week 1 to 1.9 uses in week 24.^40^ Notably, those aged 46-60 years were significantly more engaged with the app based on days of on-demand use (0.48 days more weekly) and daily on-demand use (1.78 uses more per day) than those aged 18-29 years, as were females compared with males.^40^

Adherence was not reported in many of the studies; however, where it was reported, it was high. In a randomized, parallel-arm study investigating the use of SlowMo, a digitally supported reasoning intervention to reduce paranoia severity, versus treatment as usual (TAU), 71.4% of 140 participants who attended at least 1 face-to-face therapy session met adherence criteria for mobile app use (ie, had at least 1 screen interaction after at least 3 face-to-face therapy sessions).^61^ Likewise, in an RCT of Early signs Monitoring to Prevent relapse in psychosis and prOmote Well-being, Engagement, and Recovery (EMPOWER), a smartphone app blended with peer and clinician support, versus TAU, 71% of participants randomized to EMPOWER met the criteria for adherence to daily monitoring (>33% daily usage) at 12 months.^56^ Adherence to the ClinTouch symptom-monitoring app was also high during an RCT versus TAU, with 84% of participants allocated to ClinTouch demonstrating acceptable adherence, defined as responding to >33% of alerts over 12 weeks.^62^ Secondary analysis of data from an 8-week RCT of internet-based cognitive behavioral therapy for psychosis (iCBTp) found that it was, however, not possible to predict adherence based on sociodemographic, psychopathological, and treatment-related variables.^63^

#### Satisfaction with DTs

Twenty studies specified how satisfied participants were with DT use,^16^^,^^20^^,^^24^^,^^39^^,^^43^^,^^45-47^^,^^49^^,^^50^^,^^52^^,^  ^54^^,^^55^^,^^60^^,^^64-69^ and, in many of these studies, the proportion of participants reporting that they were satisfied with the DTs was high (68%-100%).^39^^,^^43^^,^^45^^,^^47^^,^^50^^,^^54^^,^^55^^,^^60^^,^^64^^,^^65^ In the Momentum RCT of a digital shared decision-making app versus TAU, participants reported being “somewhat satisfied” with the app at the 6-month assessment, based on a mean score (6.4 out of 12) of the study’s App Rating Questionnaire.^52^ Several studies reported that participants found the DTs easy to use (FOCUS: 87.5% of participants; m-RESIST [Mobile Therapeutic Attention for Patients with Treatment Resistant Schizophrenia]: >80% of participants; Heal Your Mind: ~83% of participants; MedActive: 100% of participants; Mobile Enhancement of Motivation in Schizophrenia [MEMS]: 96% of participants),^43^^,^^45-47^^,^^60^ were confident using them (FOCUS: 90.3% of participants),^60^ and liked the idea of weekly monitoring via mobile phone (HEINS, an internet-based mobile intervention for use after inpatient treatment: 100% of participants).^24^ Other positive aspects of DTs noted by participants included ready access to information,^70^ the opportunity for self-reflection,^71^ and the helpfulness of regular reminders for daily tasks and medical appointments.^72^ Furthermore, in several studies, participants stated that they would recommend the DT to others.^20^^,^^43^^,^^54^^,^^55^

#### Effective Usage

Effective usage was difficult to quantify, but where reported, studies were generally favorable. For example, in a survey of young people with early psychosis who had used the Heal Your Mind app, a smartphone app for cognitive behavioral case management and symptom monitoring, overuse or misuse of the app was not observed and there were no reports of overuse or poorly controlled usage, suggesting that people with schizophrenia can use this DT effectively.^45^ Participants who completed the non-comparative 12-week Mobile Assessment and Treatment for Schizophrenia (MATS) study had an average valid response rate for outcome assessment questions of 86%, and 86% of phones were returned undamaged.^73^ In the 14-day feasibility study of the Motivation and Skills Support (MASS) app, participants reported low ratings of difficulty with program use.^67^ Similarly, in the RCT of MEMS plus goal setting versus goal setting alone, 92% of participants in the MEMS group reported that the motivational texts were useful, helped their motivation, and helped them to reach their goals, results that are supportive of effective use.^47^

### RQ2: What is the efficacy/effectiveness of DTs in schizophrenia/SSD?

Of the 24 studies relevant to RQ2, 16 were comparative studies and 8 were non-comparative studies (Table 1 and Suppl. Tables 5 and 6). Nine of the 16 comparative studies^20^^,^^44^^,^^47^^,^^50^^,^^54^^,^^56^^,^^61^^,^^74^^,^^75^ and all 8 non-comparative studies^17^^,^^18^^,^^59^^,^^60^^,^^65^^,^^66^^,^^73^^,^^76^ reported significant improvement in ≥1 efficacy outcome versus a comparator or baseline value, respectively (Table 1). All 24 studies reported ≥1 efficacy outcome that did not show significant improvement with DT use (Suppl. Table 6).

**Table 1:** Outcomes with significant differences between groups (comparative studies) or between pre- and post-intervention (non-comparative studies) for Research Question 2: *What is the efficacy/effectiveness of DTs in schizophrenia/SSD?*

**Comparative studies**								
**DT**	**Control**		**Outcomes with significant between-group difference (DT versus control)**					
		**Negative symptoms**	**Positive symptoms**	**Cognitive performance**	**Mood, depression and/or anxiety**	**Medication adherence**	**Recovery**	**General/other symptoms**
SAVVy + TAU^20^	TAU							✓ (2)^a^
T4RP^74^	TAU		✓^b^			✓^b^		
SlowMo + TAU^61^	TAU		✓(8 [3^a^ + 5^b^])		✓^a^			
EMPOWER^56^	TAU	✓^b^			✓^a^	✓^a^		
MCI-S mobile app + weekly monitoring^75^	MCI-S mobile app	✓^b^	✓^b^					✓(2)^b^
SMARTapp + personalized feedback^44^	SMARTapp only		✓^a^^,^^c^					
MEMS + goal setting^47^	Goal setting	✓(3)^b^						
PRIME^50^	WL + TAU	✓(2)^a^			✓^a^			
iCBTp+ TAU^54^	WL + TAU		✓^a^^,^^d^					
**Non-comparative studies**								
**DT**			**Outcomes demonstrating significant difference between pre- and post-DT intervention**					
		**Negative symptoms**	**Positive symptoms**	**Cognitive performance**	**Mood, depression and/or anxiety**	**Medication adherence**	**Recovery**	**General/other symptoms**
FOCUS^60^			✓^b^		✓^a^			✓(2)^b^
Self-management mobile app (unbranded)^18^			✓^b^					
weCOPE^76^							✓(8)^a^	✓^b^
MASS^17^			✓^b^					
CBT2go^59^		✓^b^	✓^b^^,^^e^					
MATS^73^			✓^a^			✓^a^		
A4i^65^			✓(2)^a^^,^^f^		✓(2)^a^^,^^f^			✓ (2)^a^^,^^f^
MACS^66^							✓^a^	✓^b^

Where there is more than 1 outcome type in a table cell, the number is given in brackets. Outcomes with no significant differences between groups (comparative studies) or between pre- and post-intervention are shown in Suppl. Table 6. Abbreviations: A4i, App4Independence; CBT, cognitive behavioral therapy; DT, digital therapeutic; EMPOWER, Early signs Monitoring to Prevent relapse in psychosis and prOmote Well-being, Engagement, and Recovery; iCBTp, Internet-based cognitive-behavioral therapy for psychosis; MACS, Mobile After-Care Support; MASS, Motivation and Skills Support; MATS, Mobile Assessment and Treatment for Schizophrenia; MCI-S, metacognitive intervention for schizophrenia; MEMS, Mobile Enhancement of Motivation in Schizophrenia; PRIME, personalized real-time intervention for motivational enhancement; SAVVy, Smartphone-Assisted coping-focused interVention for Voices; SMARTapp, Schizophrenia Mobile Assessment and Real-Time feedback app; T4RP, texting for relapse prevention; TAU, treatment as usual; WL, wait list.

a Self-reported.

b Clinician/researcher/interviewer assessed.

c Significant improvements in positive symptoms were seen in both the intervention and control arms.

d Intention-to-treat population.

e Significance was not observed at all time points assessed.

f After controlling for gender, age, and baseline symptomatology.

#### Significant Findings

##### Negative Symptoms

Negative symptoms were significantly improved in 4 comparative studies and 1 non-comparative study.^47^^,^^50^^,^^56^^,^^59^^,^^75^ DTs associated with significant improvements in negative symptoms included EMPOWER, metacognitive intervention for schizophrenia (MCI-S) mobile app plus weekly monitoring, MEMS plus goal setting, personalized real-time intervention for motivational enhancement (PRIME), and CBT2go.^47^^,^^50^^,^^56^^,^  ^59^^,^^75^ Use of EMPOWER for up to 12 months or MCI-S for 10 weeks was significantly associated with improvements in the Positive and Negative Syndrome Scale (PANSS) negative score versus TAU at 12 months or baseline, respectively.^56^^,^^75^ Mobile Enhancement of Motivation in Schizophrenia significantly improved Clinical Assessment Interview for Negative Symptoms (CAINS) motivation and anticipatory pleasure scores, and the motivation item of the Quality of Life scale (QLS) over 8 weeks versus goal setting alone in people with SSD.^47^ Additionally, CBT2go significantly improved experiential negative symptoms on the CAINS motivation and pleasure (CAINS-MAP) subscale at the end of the 24-week study period relative to baseline.^59^

##### Positive Symptoms

Positive symptoms were significantly improved in 5 comparative and 6 non-comparative studies.^17^^,^^18^^,^^44^^,^^54^^,^^59-61^^,^^65^^,^^73-75^ T4RP (a text-messaging program targeting early warning symptoms of relapse) significantly improved PANSS positive score over 6 months versus TAU in an RCT of people with schizophrenia and schizoaffective disorder.^74^ SlowMo plus TAU also significantly improved positive symptoms versus TAU over 24 weeks, as assessed by multiple outcomes, including the Green et al. Paranoid Thoughts Scale (GPTS) Part B score, revised GPTS (R-GPTS) total and persecution scores, Psychotic Symptom Rating Scales (PSYRATS) total, distress and conviction scores, and Scale for the Assessment of Positive Symptoms (SAPS) persecutory delusions and ideas and delusions of reference scores.^61^ Significant improvements in positive symptoms measured by Community Assessment of Psychic Experiences (CAPE) positive score were also reported after 3 weeks among people with SSD randomized to receive Schizophrenia Mobile Assessment and Real-Time feedback app (SMARTapp) feedback according to their daily ESM questionnaire entries and in those for whom the SMARTapp included only ESM questionnaires without personalized feedback.^44^ People with SSD with access to a CBT-based, internet-based self-help platform and an optional smartphone app also saw significant improvements in positive symptoms over 8 weeks compared with those who were on a waiting list and did not have access to the intervention, as measured by the Launay–Slade Hallucination Scale (LSHS).^54^ These significant improvements continued at the 6-month follow-up.^54^

##### Cognitive Performance

No studies reported significant improvements in cognitive performance.

##### Mood, Depression, and/or Anxiety

Mood, depression, and/or anxiety were significantly improved in 3 comparative and 2 non-comparative studies.^50^^,^^56^^,^^60^^,^^61^^,^^65^ Penn State Worry Questionnaire (PSWQ) scores were significantly improved with the use of SlowMo + TAU versus TAU alone over 12 and 24 weeks.^61^ Additionally, use of EMPOWER significantly improved depression scores on the Hospital Anxiety and Depression Scale (HADS) versus TAU at 12 months.^56^ The Beck Depression Inventory (BDI)-II score was significantly improved with use of PRIME for 12 weeks versus TAU, an improvement that was maintained 3 months post-trial.^50^ Use of FOCUS over a 1-month period also significantly improved BDI-II scores in people with schizophrenia or SSD.^60^ Finally, those using the A4i platform for 1 month saw significant improvements in the depression and phobic anxiety domains of the BSI.^65^

##### Medication Adherence

Medication adherence was significantly improved in 2 comparative studies and 1 non-comparative study.^56^^,^^73^^,^^74^ An RCT of T4RP versus TAU showed significant improvements in injectable medication adherence over 6 months.^74^ Use of EMPOWER for 12 months also significantly improved scores on the Medication Adherence Report Scale (MARS) versus TAU alone,^56^ and participants in the non-comparative 12-week MATS study saw improved medication adherence during the study.^73^

##### Recovery

Recovery was significantly improved in 2 non-comparative studies.^66^^,^^76^ Those using the weCOPE app showed significant improvements from baseline to Week 8 in the Recovery Assessment Scale (RAS) total score and several of its items (personal confidence and hope, goals and success orientation, and life beyond symptoms), in addition to significant improvements in Empowerment Scale total score and its dimensions (eg, self-esteem and confidence, wrath, and optimism).^76^

##### General/other Symptoms

Significant improvements in general/other symptoms were seen in 2 comparative studies and 4 non-comparative studies.^20^^,^^60^^,^^65^^,^^66^^,^^75^^,^^76^ Improved confidence in coping with voices day-to-day and awareness of patterns in voices were significantly improved over 8 weeks among people randomized to Smartphone-Assisted coping-focused interVention for Voices (SAVVy) with TAU versus TAU alone in the RCT of people with persisting and distressing voices.^20^ Metacognitive intervention for schizophrenia plus weekly mentoring sessions significantly improved PANSS total and general psychopathology scores over 10 weeks compared with the app-based intervention alone, as did use of the FOCUS smartphone intervention over a 1-month period.^60^^,^^75^ Furthermore, use of weCOPE, a smartphone app for symptom monitoring, problem-solving, anxiety management, and goal setting, significantly improved PANSS general psychopathology at 8 weeks compared with baseline.^76^ Additionally, use of App4Independence (A4i) (a digital platform consisting of a mobile app and a connected clinician/case manager portal) and the app-based intervention Mobile After-Care Support (MACS) were associated with improvements in general/other symptoms after 1 month, as assessed by the Brief Symptom Inventory (BSI) and the Brief Psychiatric Rating Scale (BPRS), respectively.^65^^,^^66^

### RQ3: What are the safety concerns associated with DTs in schizophrenia/SSD?

Many studies did not report any information on safety or adverse events (AEs). Of the 38 papers assessed, 9 comparative and 3 non-comparative studies included safety data but not in a consistently detailed manner^20^^,^^43^^,^^45^^,^^48^^,^^54^^,^^56^^,^^61^^,^^62^^,^^64^^,^^65^^,^^77^; for example, some studies did not specify the AEs, or provide intensity or resolution status (Table 2 and Suppl. Table 7). Of the 12 studies with safety data, 10 reported ≥1 AE, such as physical AEs, admission to psychiatric hospital during follow-up, or referral to crisis care.^20^^,^^43^^,^^54^^,^^56^^,^^61^^,^^62^^,^^64^^,^^65^^,^^77^^,^^78^

**Table 2:** Safety findings for Research Question 3: *What are the safety concerns associated with DTs in schizophrenia/SSD?*

**Comparative studies**				
**DT**	**Control**	**AEs**		**Safety summary**
		**In DT group**	**In control group**	
SAVVy + TAU^20^	TAU	1 hospital admission (SAE)	1 hospital admission (SAE)	Both SAEs unrelated to trial or intervention.
FOCUS^78^	TAU	1 all-cause death		No other AEs or SAEs reported.
CBT2go^77^	Self-monitoring TAU	10 hospitalizations	Self-monitoring: 12 hospitalizations TAU: 9 hospitalizations	AEs unlikely to be related to study interventions.
SlowMo + TAU^61^	TAU	25 SAEs	26 SAEs	In the TAU group, 1 SAE was definitely related to trial participation. In the SlowMo group, 1 SAE was possibly related to trial participation and 1 SAE was unlikely related to trial participation. All other SAEs were not related to trial participation. 54 AEs; common AEs included hospitalization during follow-up (SlowMo: *n* = 8, TAU: *n* = 10), referral to crisis care, (SlowMo: *n* = 5, TAU: *n* = 2), physical (SlowMo: *n* = 8, TAU: *n* = 2), other (SlowMo: *n* = 5, TAU: *n* = 4).
PEAR-004 + TAU^64^	Digital sham + TAU	12 AEs	10 AEs 1 SAE	All reported AEs were mild or moderate. Most AEs were not suspected to be related to treatment. The SAE was not suspected to be related to treatment. Most AEs were resolved or recovering at study end.
EMPOWER^56^	TAU	29 AEs (13 app-related) 11 SAEs (1 app-related) 1 death	25 AEs 15 SAEs 0 deaths	The 1 death was not considered related to use of the app. Most AEs were severe. The app-related AE was a hospitalization, in part, related to feeling overwhelmed at the point of app installation. Aside from AEs related to the app, there were no AEs related to the device.
ClinTouch + TAU^62^	TAU	3 AEs	NR	AEs: increased anxiety prompted by questions; increased irritation due to the alert beeps; 1 charger exploded. All 3 participants continued trial to completion.
Florence + TAU^48^	TAU	0	0	No harmful effects were observed as a result of the trial.
iCBTp + TAU^54^	WL + TAU	3 AEs	8 AEs	1 event in the iCBTp group potentially related to the study (experience of a vision while filling in a questionnaire). 2 events in the control group potentially related to the study (change in antipsychotic medication [*n* = 2]). The most common negative effect (reported in 38% of participants) was the absence of human contact in the self-help internet program.
**Non-comparative studies**				
**DT**		**AEs**		**Safety summary**
m-RESIST^43^		3 SAEs requiring hospitalization		SAEs had no direct association with intervention or protocol procedures.
A4i^65^		1 report of anxiety regarding text messages		No AEs observed aside from the report of anxiety.
HYM^45^		0 AEs related to app use		95.8% of participants did not find their symptoms exacerbated by app use.

Abbreviations: A4i, App4Independence; AE, adverse event; CBT, cognitive behavioral therapy; DT, digital therapeutic; EMPOWER, Early signs Monitoring to Prevent relapse in psychosis and prOmote Well-being, Engagement, and Recovery; HYM, Heal Your Mind smartphone app; iCBTp, Internet-based cognitive-behavioral therapy for psychosis; m-RESIST, Mobile Therapeutic Attention for Patients with Treatment Resistant Schizophrenia; NR, not reported; SAVVy, Smartphone-Assisted coping-focused interVention for Voices; SAE, serious adverse event; TAU, treatment as usual; WL, wait list.

Three RCTs (EMPOWER, ClinTouch [a symptom monitoring app], and iCBTp reported some AEs to be related or potentially related to intervention or trial participation.^54^^,^^56^^,^^62^ The non-comparative study of A4i reported an ambiguous relationship of an AE to the intervention (a participant attributed their anxiety to the app prompt questions but also stated that, if it was available, they would use the app in the future).^65^ While not labeled as an AE, but reported within a questionnaire on side-effects, the iCBTp study stated the most common negative effect (reported in 38% of participants) was the absence of human contact in the self-help internet program.^54^

Five studies reported serious AEs (SAEs), such as suicidal ideation and hospitalization due to worsening of psychotic symptoms.^20^^,^^43^^,^^56^^,^^61^^,^^64^ Of these 5 studies, 3 (SAVVy, PEAR-004, m-RESIST) did not consider any of the reported SAEs to be related to DT use or trial participation.^20^^,^^43^^,^^64^ In the EMPOWER study, 1 SAE (hospital admission) was considered related to the app as it was, in part, related to feeling overwhelmed at the point of app installation.^56^ In the SlowMo study (*N* = 362), 1 of 26 SAEs in the control group (TAU) was definitely related to trial participation, while 1 of 25 SAEs in the SlowMo group was possibly related to trial participation and 1 was unlikely related to trial participation; all other SAEs were not related to trial participation.^61^

### RQ4: What is the impact of DTs in schizophrenia/SSD on real-world functioning, QoL, and other outcomes?

In total, 16 comparative and 8 non-comparative studies included data relevant to RQ4 (Table 3 and Suppl. Tables 8 and 9). Seven of the comparative and 4 of the non-comparative studies reported significant improvement in ≥1 outcome related to functioning, QoL, patient–provider engagement, patient insight, competency/self-efficacy, and/or other outcomes (Table 3).^17^^,^^50^^,^^52^^,^^54^^,^^56^^,^^59^^,^^61^^,^^73^^,^  ^75-77^ All 24 studies included ≥1 outcome that did not show significant improvement with DT use (Suppl. Table 9).

**Table 3:** Outcomes with significant differences between groups (comparative studies) or between pre- and post-intervention (non-comparative studies) for Research Question 4: *What is the impact of DTs in schizophrenia/SSD on real-world functioning, quality of life, and other outcomes?*

**Comparative studies**											
**DT**	**Control**	**Outcomes with significant between-group difference (DT versus control)**									
		**Functioning**	**QoL**	**HCRU**	**Stigma**	**Patient–provider engagement**	**Insight**	**Competency/self-efficacy**	**Reward responsivity**	**Medication beliefs**	**Other outcomes**
CBT2go^77^	TAU	✓^a^									
SlowMo + TAU^61^	TAU		✓ (2 [1^a^ + 1^b^])								✓(4)^b^
EMPOWER^56^	TAU					✓^c^	✓^b^				
MCI-S mobile app + weekly mentoring^75^	MCI-S mobile app	✓^a^					✓ (2)^b^				
PRIME^50^	WL + TAU							✓^b^			✓^b^
Smartphone app + TAU^52^	TAU					✓ (3)^a^^,^^b^^,^^d^					
iCBTp + TAU^54^	WL + TAU							✓^b^^,^^e^			
**Non-comparative studies**											
**DT**		**Outcomes demonstrating significant difference between pre-and post-DT intervention**									
		**Functioning**	**QoL**	**HCRU**	**Stigma**	**Patient–provider engagement**	**Insight**	**Competency/self-efficacy**	**Reward responsivity**	**Medication beliefs**	**Other outcomes**
weCOPE^76^		✓^a^	✓ (3)^b^					✓ (3)^b^			
MASS^17^		✓^b^									
MATS^73^		✓^b^									
CBT2go^59^		✓^b^^,^^f^	✓^a^^,^^f^								✓^b^

Outcomes with no significant differences between groups (comparative studies) or between pre- and post-intervention are shown in Suppl. Table 9. Abbreviations: CBT, cognitive behavioral therapy; DT, digital therapeutic; EMPOWER, Early signs Monitoring to Prevent relapse in psychosis and prOmote Well-being, Engagement, and Recovery; HCRU, healthcare resource utilization; iCBTp, internet-based CBT for psychosis; MASS, Motivation and Skills Support; MATS, Mobile Assessment and Treatment for Schizophrenia; MCI-S, metacognitive intervention for schizophrenia; PRIME, personalized real-time intervention for motivational enhancement; QoL, quality of life; TAU, treatment as usual; WL, wait list.

a Clinician/researcher/interviewer-administered assessment.

b Self-reported.

c Carer-assessed.

d Based on patient records/data.

e Intention-to-treat population.

f Significance was not observed at all time points assessed. Where there is more than 1 outcome type in a table cell, the number is given in brackets.

#### Significant Findings

##### Functioning

Functioning was significantly improved in 2 comparative and 4 non-comparative studies.^17^^,^^59^^,^^73^^,^^75-77^ Digital therapeutics that were associated with significant improvements in functioning versus control or baseline were CBT2go, MCI-S mobile app use with weekly mentoring sessions, weCOPE, MASS, MATS, and MACS.^17^^,^^59^^,^^73^^,^^75-77^ CBT2go use was associated with significant improvements in community functioning, measured by the Specific Level of Functioning (SLOF) scale score, compared with TAU, in a 24-week RCT.^77^ Improvements in Personal and Social Performance Scale (PSP) total scores were also observed at 8 weeks versus baseline among participants using weCOPE^76^ and over 10 weeks among participants receiving weekly contact mentoring sessions and who had access to the MCI-S mobile app, developed for symptom relief and functional improvements in individuals with schizophrenia.^75^ However, there were no significant improvements in PSP total scores over 10 weeks in those who did not receive the weekly mentoring sessions and received the app-based intervention only or in any of the PSP subscale scores in either of the treatment groups.^75^ The smartphone app MASS, designed with stakeholder input to target social motivation and social goal attainment, also significantly improved functioning (measured by the Social Functioning Scale [SFS]) in participants after 8 weeks of use; however, this improvement was not maintained after 3 months of follow-up.^17^ Negative beliefs about socializing were also significantly reduced among participants of the MATS study, who received daily interactive text messages for up to 12 weeks to encourage social activity and connection with others.^73^

##### Quality of Life

Quality of life was significantly improved in 1 comparative and 2 non-comparative studies.^59^^,^^61^^,^^76^ Significant improvements in QoL were reported in the SlowMo RCT for app users versus participants receiving TAU at week 24, as measured by the Manchester Short Assessment of Quality of Life (MANSA) and the Warwick–Edinburgh Mental Wellbeing Scale (WEMWBS).^61^ Users of weCOPE saw significant improvements in 3 domains of social satisfaction at 8 weeks compared with baseline,^76^ and improvements in Abbreviated QoL Scale (A-QLS) scores were observed from baseline to 12 weeks with CBT2go, although improvements with CBT2go were not maintained at 18 and 24 weeks.^59^

##### Patient–provider Engagement

Significant improvements in the level of engagement between patients and care providers were seen in 2 comparative studies.^52^^,^^56^ In the RCT of participants using a shared decision-making app as a supplement to TAU for up to 6 months, significant improvements were seen in self-perceived patient activation, confidence in communications and interactions with care provider, and feeling prepared to make treatment-related decisions compared with participants receiving TAU alone.^52^ Early signs Monitoring to Prevent relapse in psychosis and prOmote Well-being, Engagement, and Recovery use also improved participants’ engagement with community mental health services versus TAU, based on significantly improved scores on the treatment-adherence domain of the Service Engagement Scale (SES) in those using EMPOWER versus TAU; however, no significant improvements between treatment groups were observed in the SES total or other domain (availability, collaboration, help seeking) scores.^56^

##### Insight

In 2 RCTs, DT use was associated with some improvements in participants’ personal beliefs about their illness (EMPOWER vs TAU),^69^ and positive beliefs about worry and regarding the need to control thoughts (MCI-S mobile app + weekly mentoring sessions vs MCI-S mobile app alone).^56^^,^^75^ In the EMPOWER study, this was based on improvements with EMPOWER versus TAU on 1 (control) of the 5 domains of the revised Personal Beliefs About Illness Questionnaire.^56^ In the MCI-S study, participants using the MCI-S app, in addition to receiving weekly mentoring sessions, demonstrated improvements from baseline to week 10 on 2 of the 6 domains of the Metacognitive Scale-30 compared with participants who were using the MCI-S app alone.^75^

##### Competency/self-efficacy

Self-efficacy outcomes were significantly improved with PRIME versus those on a wait list receiving TAU, and post- vs pretreatment with weCOPE.^50^^,^^76^ Significant improvements were also seen in participants’ psychological resources for those accessing iCBTp versus those on a wait list receiving TAU, based on improvements on the Mindful Attention and Awareness Scale.^54^ However, there were no improvements between groups in self-esteem and social skills (other measures of psychological resources), as measured by the Rosenberg Self-Esteem Scale and the Brief Interpersonal Competence Questionnaire, respectively.^54^

##### Other Outcomes

Other outcomes were significantly improved in 2 comparative studies and 1 non-comparative study.^50^^,^^59^^,^^61^; these outcomes were related to defeatist beliefs (PRIME, CBT2go),^50^^,^^59^ beliefs about self (SlowMo),^61^ and reasoning processes pertaining to fast (intuitive) and slow (analytic) thinking (SlowMo).^61^

##### Healthcare Resource Utilization, Reward Responsivity, Medication beliefs, and Stigma

There were no reports of significant improvements in healthcare resource utilization (HCRU), reward responsivity, medication beliefs, or stigma outcomes with DT use in any of the studies assessed (Table 3 and Suppl. Table 9).

## Discussion

The findings of this SLR of DTs for people with SSD portray a varied and evolving landscape. Many of the DTs for this population are in early-stage development and may continue to be developed and refined over time, based on further studies and user feedback. The DTs reviewed differ somewhat in their aims and functionality, and DT studies varied in terms of their study designs, outcomes, and assessments. As such, this review provides a useful resource with which to begin to determine how best to assess DT use, efficacy/effectiveness, and safety in this population.

Across the included studies, participants were receptive to support for managing their condition via a DT; participants demonstrated high levels of engagement through responsiveness to DT functions (eg, app prompts) and were generally satisfied or positive toward using DTs.^16^^,^^17^^,^^20^^,^^24^^,^^39-56^^,^^60-62^^,^^64-67^^,^^73^ Digital therapeutics engagement and/or adherence was generally over 50%.^20^^,^^24^^,^^39-42^^,^^44^^,^^46-53^^,^^55^^,^^58^^,^^61^^,^^62^^,^^65^ These engagement/adherence levels, which were based on study durations of 2 weeks to 12 months,^17^^,^^20^^,^^24^^,^^39-55^ appear to be similar to antipsychotic adherence levels, which reportedly range from 11% to 80% based on studies of 1 to ~3 years, or based on people’s medical history or information from caregivers.^79-82^ Of note, there was variation in how DT engagement and adherence were defined across studies. The most common definition of adherence, used in 3 of the studies, was a >33% cut-off point for adherence (eg, responding to >33% of alerts over 12 weeks,^62^ or >33% daily usage of daily monitoring at 12 months,^56^ or having ≥1 screen interaction after ≥3 face-to-face therapy sessions).^61^ The absence of a standard methodology for assessing and reporting adequate engagement or adherence in DT studies made it challenging to interpret and report this outcome in a consistent manner.

Although there may be concerns DT use could prove challenging for people with schizophrenia, given cognitive impairment is a key feature of the condition^83^ and people with schizophrenia generally have lower reading ability compared with others of a similar age without schizophrenia,^84^ findings from this SLR indicate participants found DTs easy to use, were confident using them,^16^^,^^39^^,^^43-47^^,^^55^^,^^56^^,^^60^^,^^66^ and were willing to integrate DTs into their daily lives in the real world.^39^ However, as this SLR found no reports of cognitive improvements in the included studies, and there was little focus on pre-study levels of cognitive impairment, it is difficult to evaluate cognition as a moderator of DT engagement. It is worth noting the findings from a previous study assessing the effect of premorbid cognitive abilities on study outcomes for a DT targeting social functioning in psychosis: Although premorbid cognitive abilities were not found to affect DT engagement, there was a weak association between higher cognitive abilities and greater improvements in the primary treatment outcome of social functioning.^85^ In addition, a recent, fully remote RCT, published after the searches for the current SLR were performed, examined the benefits of delivering targeted cognitive and social cognitive training (TCT) in conjunction with the PRIME app, an intervention targeting motivated behavior.^86^ The study found that adding TCT alongside PRIME improved global cognition (although not significantly) and significantly improved motivational impairments relative to control conditions (when PRIME was used with computer games instead of TCT).^86^ Therefore, some outcomes may be more responsive in participants with strong cognitive skills, and this is likely worthy of future study.

Building on the high levels of engagement reported across the studies, there were many significant improvements in a variety of outcomes, such as improvements in positive, negative, and general/other symptoms; depression/anxiety; medication adherence; recovery; functioning; and QoL, as well as other outcomes.^17^^,^^18^^,^^20^^,^^44^^,^^47^^,^^50^^,^^52^^,^^54^^,^^56^^,^^59-61^^,^^65^^,^^66^^,^^73-77^ However, most studies included a mix of significant and non-significant findings. While this will, in part, reflect the DT under evaluation, other contributing factors must be considered. Firstly, due to heterogeneity in study design and methodology, outcomes were not uniformly categorized into primary and secondary endpoints; many studies did not specify outcome hierarchy. DT outcomes were also not reviewed by type of study design or whether they were used as a standalone treatment or blended with in-person therapy. It therefore needs to be acknowledged that the number of significant and non-significant outcomes was an overall observation, not a conclusive statement on efficacy reflecting outcomes of equal weighting. For example, outcomes may have been non-significant if they were not related to the primary treatment target of the DT or because of limitations in the study design. Secondly, the control group appears to be an important design consideration. Sixteen of the included studies were RCTs, in which TAU was commonly used as a control; 11 of these studies demonstrated efficacy/effectiveness in improving symptoms, functioning, QoL, or other outcomes.^20^^,^^44^^,^^47^^,^^50^^,^^52^^,^^54^^,^^56^^,^^61^^,^^74^^,^^75^^,^^77^ However, in the PEAR-004 study, in which a digital sham was used as a control, the primary endpoint (change in total PANSS score) was not met, possibly related to the design of the sham control.^64^ For example, post-study interviews demonstrated that the sham app may have had unintended benefits (eg, served as a mindfulness exercise that distracted participants from their symptoms).^64^ In another study with a digital sham control, published after the searches for the current SLR were performed, there were no significant differences between the DT and control groups for any of the primary or secondary outcomes.^87^ Together, these findings highlight the need for caution when interpreting efficacy results from studies comparing a digital intervention against TAU where it is not possible to assess whether the DT itself or simply the presence of a digital intervention is the reason for improvement. A third consideration is the outcomes assessed in these studies. Targeting and assessing positive symptoms, which may reduce over time,^88^ may be less suitable for a DT study than a traditional antipsychotic study. Instead, DT studies could be designed to target negative symptoms and functioning, given that DT content is often geared toward motivation and goal attainment, both of which are less likely to reduce over time.^88^^,^^89^ Fourth, study duration should be considered; this varied from 2 weeks to 2 years across the included publications. While this is similar to the reported median duration of antipsychotic studies (6 weeks [range: 3-28 weeks]),^90^ in general, shorter studies, such as the 1-month FOCUS study,^39^ are designed primarily to assess DT usability rather than efficacy. Fifth, many of the studies are likely to have compensated individuals for trial participation, either financially (eg, $20/hour to complete assessments in the PRIME study)^50^ or through providing a commodity (eg, free calls, text, and internet during the FOCUS trial).^60^ This may have acted as an incentive to use the app. Finally, the heterogeneity of the DTs reviewed should be noted. Some DTs, for example, are designed to provide the user with tools/strategies for managing psychosis, some are aimed at goal setting or increasing social functioning, and others are intended for relapse prevention or to assist cognitive restructuring. As such, it is difficult to compare efficacy outcomes across studies with broadly different characteristics.

Eleven of 24 studies reported significant improvement in ≥1 outcome relating to functioning, QoL, or other aspect (eg, insight and self-efficacy), and all 24 studies included ≥1 outcome that did not demonstrate a significant improvement with DT use. The high proportion of studies with ≥1 non-significant functioning/QoL outcome could reflect that many of the functioning and QoL assessments used in the studies are insensitive to subtle improvements that may occur with DT use, particularly over a short study period. These findings may also reflect a floor effect, with many people whose condition is already substantially affecting daily functioning finding improved outcomes difficult without more comprehensive and intensive treatment and support (eg, addition of rehabilitation programs, such as supported employment and education).^91^^,^^92^

Few studies comprehensively assessed DT safety. The most commonly reported AE or SAE occurring during the studies was hospitalization, with most not considered related to the DT.^20^^,^^43^^,^^61^^,^^77^ Notably, safety outcomes were only available in a third of the papers assessed, and safety was not uniformly reported. This variability in approach, and the need for best-practice routine monitoring and evaluation, was also recognized in a systematic review of AEs in DTs for psychosis.^93^ The review found significant variability in data quality, with ~10% of AE reports classified as unclear/insufficiently detailed and 27% of studies not reporting relatedness of AE to DT or study participation.^93^ In addition, AE data were only shared by 20% of authors when asked, indicating restricted data reporting may be common in DT studies.^93^

In terms of how this SLR complements other research in this field, while several reviews and meta-analyses exist on the topics of acceptability and efficacy of digital health applications in schizophrenia, the literature search for this SLR was run up to January 28, 2024, thereby building on the previous works of other SLRs, such as that of Alvarez-Jimenez et al. (searches run up to August 2013),^94^ Firth et al. (to May 2015),^95^ Chivilgina et al. (to October 2019),^96^ Loh et al. (to September 2022),^97^ and Smith et al. (to September 2023).^98^ However, unlike some articles,^94-99^ this SLR did not include health/wellbeing technologies (ie, direct to consumer apps), ecological momentary assessments alone, web-based psychoeducation, social networking, patient-to-expert communication, or virtual reality interventions, nor did it limit its scope of research to a particular symptom (eg, psychosis), thus providing a body of work pertinent to the diagnoses of SSD, schizophrenia, and schizoaffective disorder while focused specifically on DTs.

Other reviews in this therapy area have taken a different approach to that used for this SLR. For example, the review by Smith et al. reported findings from a literature search and multidisciplinary group held to reach consensus on the challenges and potential solutions to collecting data and delivering treatment with digital mental health technologies in schizophrenia and severe mental illness.^98^ They identified several recommendations, such as new approaches to research, funding, and the implementation of digital mental health in real-world settings, in addition to a greater awareness of ethical issues.^98^ Chivilgina et al. also report on the ethical challenges of digital technologies for individuals with schizophrenia, noting a lack of ethical considerations from their literature search.^22^^,^^96^ Another review that took a different approach to that of this SLR was by Arnautovska et al.,^100^ who assessed whether digital interventions for people with schizophrenia were more effective if they were user-led or provided with human support. Just under half of the studies they assessed included human support with the digital intervention, and only 1 study showed superiority in global and social cognition outcomes when the intervention was used in conjunction with human support.^100^

Strengths of this SLR include the variety of sources used to identify eligible studies and that no limits were placed on publication year. However, limitations include widely variable study designs and durations, population sizes and characteristics, outcomes and tools used to assess them, as well as the heterogeneity of DTs, their aims and functionality. The literature search excluded computerized cognitive training, and no formal quality assessment of the individual studies was conducted, nor was there an assessment of level of facilitator/care team intervention. Furthermore, while this SLR focused on SSDs as patients with an SSD diagnosis have somewhat different symptoms, care engagement patterns, and treatment needs than patients with other severe mental disorders, not all people across the studies had a diagnosis of SSD. Inclusion criteria stipulated that studies including populations with a mix of diagnoses (or in which individuals experienced a first episode or early psychosis) were included if ≥75% of the population were diagnosed with schizophrenia, schizoaffective disorder, or another SSD. The ≥75% cut-off was used as an indicator of majority, based on the premise that at any lower than 75% it could not be assumed that the reported outcomes would be applicable to the SSD population. Given studies may have included up to one-quarter of patients without schizophrenia, schizoaffective disorder, or another SSD, the results may also apply, to a degree, to a small subgroup of patients with non-SSDs. Finally, the review included data available in the public domain only, and study teams were not contacted for further data where they were missing or unpublished.^22^^,^^96^

Based on the findings of this SLR, recommendations for future studies of DTs include working toward consistency in how adequate DT engagement or adherence are defined and reported, especially given the fact that adherence can be problematic for some patients and that inadequate adherence to treatments, including to DTs, may negatively impact outcomes. Future studies should also investigate the effect of blended vs standalone interventions and different baseline patient characteristics (eg, level of symptoms, medication adherence, functioning, quality of life) may have on engagement or adherence with DTs and study outcomes. Additionally, incorporating a digital sham control into the study design is suggested to help determine whether improvements are due to the DT itself or simply due to the presence of a digital intervention. Finally, it would be beneficial for future DT studies to include a comprehensive evaluation of safety as standard practice, so that any potential harms can be monitored and assessed.

## Conclusion

This SLR provides valuable, preliminary evidence supporting DTs as an appropriate means of delivering accessible, generally safe, evidence-based care to people with SSD. Some of the DTs assessed in this review may in time become PDTs and, should they do so, awareness of these treatment modalities may need to be increased among healthcare professionals. As the development of DTs can be challenging, this review may aid future DT study design, underlining suitable outcomes and control arms to use and highlighting the need for a consistent assessment of DT safety.

## Supplementary Material

Supplementary_materials_sbaf134

## Data Availability

As a systematic literature review article, a data sharing statement is not applicable. The protocol can be found on the PROSPERO register: https://www.crd.york.ac.uk/PROSPERO/view/CRD42023476545.
