# Supplementary material for: Digital Therapeutics for People with Schizophrenia Spectrum Disorders: A Systematic Literature Review of Their Effect on Symptoms and Functioning
Source: Schizophr Bull. 2025 Aug 25;52(4):sbaf134. doi: 10.1093/schbul/sbaf134 (PMC13391626; doi:10.1093/schbul/sbaf134)
Supplement: Supplementary_materials_sbaf134 [file supplementary_materials_sbaf134.docx]

# SUPPLEMENTARY MATERIALS

*Digital therapeutics for people with schizophrenia spectrum disorders: A systematic literature review of their effect on symptoms and functioning*

Daniel Fulford, Christoph U. Correll, Philip D. Harvey, Alex S. Cohen

*Schizophrenia Bulletin*

**Contents**

**Suppl. Table 1**. Literature database searches and number of hits for (A) MEDLINE and Embase, (B) Cochrane Database of Systematic Reviews, (C) PsycINFO, and (D) additional intervention of interest via MEDLINE, Embase, Cochrane Database of Systematic Reviews and PsycINFO

Suppl. Table 2. Additional search terms for (A) conference abstracts and (B) clinical registries

**Suppl. Table 3**. Descriptions of DTs and characteristics of included studies

Suppl. Table 4. Studies relevant to Research Question 1: *Do people with schizophrenia/SSD use DTs and are they capable of using them effectively?*

**Suppl. Table 5**. Studies relevant to Research Question 2: *What is the efficacy/effectiveness of DTs in schizophrenia/SSD?*

**Suppl. Table 6**. Outcomes with no significant differences between groups (comparative studies) or between pre- and post-intervention (non-comparative studies) for Research Question 2: *What is the efficacy/effectiveness of DTs in schizophrenia/SSD?*

**Suppl. Table 7**. Studies relevant to Research Question 3: *What are the safety concerns associated with DTs in schizophrenia/SSD?*

**Suppl. Table 8**. Studies relevant to Research Question 4: *What is the impact of DTs in schizophrenia/SSD on real-world functioning, quality of life, and other outcomes?*

**Suppl. Table 9**. Outcomes with no significant differences between groups (comparative studies) or between pre- and post-intervention (non-comparative studies) for Research Question 4: *What is the impact of DTs in schizophrenia/SSD on real-world functioning, quality of life, and other outcomes?*

**References**

**Appendix.** Abbreviations

#

#### Suppl. Table 1. Literature database searches and number of hits for (A) MEDLINE and Embase, (B) Cochrane Database of Systematic Reviews, (C) PsycINFO, and (D) additional intervention of interest via MEDLINE, Embase, Cochrane Database of Systematic Reviews and PsycINFO

**(A)**

| No. | Search strategy for MEDLINE/Embase via Embase.com | No. of hits |
| --- | --- | --- |
| #1 | ‘schizophrenia’/exp OR ‘schizophrenia’ OR ‘schizoaffective psychosis’/exp OR ‘schizoaffective’ OR ‘schizo affective’ | 261943 |
| #2 | digital NEXT/1 (therapeutic OR therapeutics OR intervention OR interventions OR healthcare OR health OR treatment OR treatments OR application OR app OR software) | 19591 |
| #3 | (smartphone OR mobile) NEXT/1 (application OR app OR software OR intervention) | 28835 |
| #4 | mHealth OR ‘mobile health’ | 18062 |
| #5 | ‘software as a medical device’ | 97 |
| #6 | ‘ecological momentary intervention’ OR emi:ab,ti | 2762 |
| #7 | #2 OR # 3 OR #4 OR #5 OR #6 | 60023 |
| #8 | ‘ct 155’ OR ct155 | 16 |
| #9 | ‘pear 004’ OR pear004 | 0 |
| #10 | Actissist | 10 |
| #11 | CBT2go | 4 |
| #12 | ‘Motivation and Skills Support’ | 1 |
| #13 | FOCUS AND smartphone AND intervention | 435 |
| #14 | NST-SSD | 0 |
| #15 | eMotiph | 0 |
| #16 | A4i OR App4Independence | 8 |
| #17 | #8 OR #9 OR #10 OR #11 OR #12 OR #13 OR #14 OR #15 OR #16 | 472 |
| #18 | #7 OR #17 | 60211 |
| #19 | #18 AND #1 | 646 |

**(B)**

| No. | Search strategy for Cochrane Database of Systematic Reviews via Cochrane library | No. of hits |
| --- | --- | --- |
| #1 | (schizophrenia):ti,ab,kw OR (schizoaffective):ti,ab,kw OR (schizo affective):ti,ab,kw | 19414 |
| #2 | MeSH descriptor: [Schizophrenia] explode all trees OR | 10088 |
| #3 | MeSH descriptor: [Psychotic Disorders] this term only | 3593 |
| #4 | #1 OR #2 OR #3 | 20963 |
| #5 | digital NEXT (therapeutic OR therapeutics OR intervention OR interventions OR healthcare OR health OR treatment OR treatments OR application OR app OR software) | 2087 |
| #6 | (smartphone OR mobile) NEXT (application OR app OR software OR intervention) | 6363 |
| #7 | mHealth OR “mobile health” | 3891 |
| #8 | “software as a medical device” | 7 |
| #9 | “ecological momentary intervention” OR emi | 602 |
| #10 | #5 OR #6 OR #7 OR #8 OR #9 | 1 |
| #11 | “ct 155” OR ct155 | 5 |
| #12 | “pear 004” OR pear004 | 2 |
| #13 | Actissist | 7 |
| #14 | CBT2go | 3 |
| #15 | “Motivation and Skills Support” | 0 |
| #16 | FOCUS AND smartphone AND intervention | 477 |
| #17 | NST-SSD | 0 |
| #18 | eMotiph | 0 |
| #19 | A4i OR App4Independence | 2 |
| #20 | #11 OR #12 OR #13 OR #14 OR #15 OR #16 OR #17 OR #18 OR #19 | 495 |
| #21 | #9 OR #19 | 11564 |
| #22 | #3 AND #20 | 167 |
|  | Restricted to Cochrane Database of Systematic Reviews | 2 |

**(C)**

| No. | Search strategy for PsycINFO via APA | No. of hits |
| --- | --- | --- |
| #1 | Keywords: schizophrenia OR schizoaffective OR “schizo affective” OR Abstract: schizophrenia OR schizoaffective OR “schizo affective” | 126,868 |
| #2 | Keywords: digital NEAR/1 (Keywords: therapeutic OR Keywords: therapeutics OR Keywords: intervention OR Keywords: interventions OR Keywords: healthcare OR Keywords: health OR Keywords: treatment OR Keywords: treatments OR Keywords: application OR Keywords: app OR Keywords: software) OR Abstract: digital NEAR/1 (Abstract: therapeutic OR Abstract: therapeutics OR Abstract: intervention OR Abstract: interventions OR Abstract: healthcare OR Abstract: health OR Abstract: treatment OR Abstract: treatments OR Abstract: application OR Abstract: app OR Abstract: software) | 12,776 |
| #3 | (Keywords: smartphone OR Keywords: mobile) NEAR/1 (Keywords: application OR Keywords: app OR Keywords: software OR Keywords: intervention) OR (Abstract: smartphone OR Abstract: mobile) NEAR/1 (Abstract: application OR Abstract: app OR Abstract: software OR Abstract: intervention) | 3,165 |
| #4 | Keywords:mHealth OR “mobile health”OR Abstract: mHealth OR “mobile health” | 3,085 |
| #5 | Keywords: “software as a medical device” OR Abstract: “software as a medical device” | 34 |
| #6 | Keywords: “ecological momentary intervention” OR Abstract: “ecological momentary intervention” OR Keywords: emi OR Abstract: emi | 393 |
| #7 | #2 OR # 3 OR #4 OR #5 OR #6 | 17,738 |
| #8 | “ct 155” OR ct155 | 0 |
| #9 | “pear 004” OR pear004 | 0 |
| #10 | Actissist | 3 |
| #11 | CBT2go | 1 |
| #12 | “Motivation and Skills Support” | 3 |
| #13 | FOCUS AND smartphone AND intervention | 140 |
| #14 | NST-SSD | 0 |
| #15 | eMotiph | 0 |
| #16 | A4i OR App4Independence | 1 |
| #17 | #8 OR #9 OR #10 OR #11 OR #12 OR #13 OR #14 OR #15 OR #16 | 147 |
| #18 | #7 OR #17 | 17,780 |
| #19 | #18 AND #1 | 399 |
| #20 | Limit to journals | 373 |

**(D)**

| No. | Search strategy for MEDLINE/Embase via Embase.com | No. of hits |
| --- | --- | --- |
| #1 | ‘schizophrenia’/exp OR ‘schizophrenia’ OR ‘schizoaffective psychosis’/exp OR ‘schizoaffective’ OR ‘schizo affective’ | 263,861 |
| #2 | “Mobile Assessment and Treatment for Schizophrenia” | 2 |
| #3 | #1 AND #2 | 2 |

| No. | Search strategy for Cochrane Database of Systematic Reviews (Cochrane library) | No. of hits |
| --- | --- | --- |
| #1 | “Mobile Assessment and Treatment for Schizophrenia” | 0 |

| No. | Search strategy for PsycINFO via APA | No. of hits |
| --- | --- | --- |
| #1 | Keywords: schizophrenia OR schizoaffective OR “schizo affective” OR Abstract: schizophrenia OR schizoaffective OR “schizo affective” | 127,509 |
| #2 | Keywords: “Mobile Assessment and Treatment for Schizophrenia” OR Abstract: “Mobile Assessment and Treatment for Schizophrenia” | 1 |
| #3 | #1 AND #2 | 1 |

To note, the identified study was also identified previously from a systematic review reference list so was not added to the PRISMA diagram.

#### **Suppl. Table 2**. Additional search terms for (A) conference abstracts and (B) clinical registries

**(A)**

| Conference | Year | Search terms used (Hits) |
| --- | --- | --- |
| American Psychiatric Association (APA) | 2022 | Digital (83) App (37) Application (27) Software (23) Smartphone (18) Mobile (19) Device (17) |
|  | 2023 | Digital (61) App (55) Application (2) Software (0) Smartphone (9) Mobile (45) Device (46) |
| Schizophrenia International Research Society (SIRS) | 2022 | Digital (47) App (31) Application (61) Software (33) Smartphone (29) Mobile (17) Device (17) |
|  | 2023 | Digital (81) App (42) Application (33) Software (23) Smartphone (11) Mobile (29) Device (20) |
| American Psychological Association  (APA via PsycINFO) | 2022 | Conference: 130th American Psychological Association Annual Convention  AND (Digital OR App OR Application OR Software OR Smartphone OR Mobile OR Device (6, none relevant) |
|  | 2023 | Conference: 131st American Psychological Association Annual Convention  AND (Digital OR App OR Application OR Software OR Smartphone OR Mobile OR Device) (0, none relevant) |
|  |  | Conference: APA OR "American Psychological Association"  AND (Digital OR App OR Application OR Software OR Smartphone OR Mobile OR Device) AND schizophrenia OR schizoaffective (4, none relevant) |

**(B)**

| Conference | Search terms used | # of trials identified |
| --- | --- | --- |
| Clinicaltrials.gov | Number of trials identified for inclusion in total | |
|  | 32 | |
|  | Number of trials identified before screening per search terms | |
|  | Condition/disease: Schizophrenia  Intervention/treatment: digital | 46 |
|  | Condition/disease: Schizophrenia  Intervention/treatment: app | 59 (incl. 15 duplications) |
|  | Condition/disease: Schizophrenia  Intervention/treatment: software | 48 (incl. 8 duplications) |
|  | Condition/disease: Schizophrenia  Intervention/treatment: smartphone | 54 (incl. 41 duplications) |
|  | Condition/disease: Schizophrenia  Intervention/treatment: mobile application | 37 (incl. 30 duplications) |
| WHO International Clinical Trials Registry Platform (https://trialsearch.who.int) | Number of trials identified for inclusion in total | |
|  | 10 | |
|  | Number of trials identified before screening per search terms | |
|  | schizophrenia AND digital | 34 |
|  | schizophrenia AND app | 27 (incl. 4 duplications) |
|  | schizophrenia AND software | 22 (incl. 3 duplications) |
|  | schizophrenia AND smartphone | 18 (incl. 9 duplications) |
|  | schizophrenia AND mobile application | 6 (incl. 3 duplications) |

####

#### Suppl. Table 3. Descriptions of DTs and characteristics of included studies

| **Digital therapeutic name** | **Description** | **Study design** | **Study duration** | **Population (number treated at baseline)** | **Diag.** | **Research question** |
| --- | --- | --- | --- | --- | --- | --- |
| **Comparative studies** | | | | | | |
| SAVVy (Smartphone-Assisted coping focused interVention for Voices) including Moisés’s app^1,2^ | A smartphone app blended with standard face-to-face therapy to improve coping with hearing voices. The app provides ecological momentary assessments (EMAs) to monitor for functional analysis and ecological momentary interventions (EMIs) to support implementation of individualized coping strategies. | RCT of SAVVy + TAU vs TAU | 8 weeks | 34 (SAVVy + TAU 17; TAU 17) | SZ & other disorders | 1, 2, 3, 4 |
|  |  | *Additional analysis: qualitative analysis of semi-structured interviews* | As above | 12 |  |  |
| FOCUS^3^ | Smartphone app designed to improve coping with psychotic symptoms, social functioning, and medication adherence | RCT of FOCUS vs TAU | Up to 12 weeks | 51 (intervention 26; TAU 25) | SZ, SZA | 2, 3, 4 |
| T4RP (Texting 4 Relapse Prevention)^4,5^ | A software program that sends regular text messages to a patient’s mobile/cell phone asking questions about symptoms, with the aim of preventing relapse | RCT of T4RP vs TAU | 6 months | 40 (intervention 28; control 12) | SZ, SZA | 1, 2, 4 |
|  |  | *Additional analysis on acceptability* |  |  |  |  |
| CBT2go^6^ | One session of in-person CBT integrated with automated thought challenging/  adaptive behavior delivered via a smartphone | RCT with 3 arms: CBT2go; self-monitoring (SM); TAU | 24 weeks | 229 (CBT2go 77; SM 69; TAU 83) | SZ, SZA, or BD | 1, 2, 3, 4 |
| HEINS (Heidelberg internet-based aftercare for patients with SSD)^7^ | Software developed to provide internet-based aftercare through the assessment and monitoring of mental health in patients with SSD. HEINS provides schizophrenia psychoeducation, access to an individual crisis plan, psychiatrist contact via internet chat/telephone, and weekly supportive monitoring. HEINS is accessible as text messaging or via smartphone/internet | RCT of TAU + HEINS vs TAU | 6 months | 25 (intervention 12; control 13) | SSD | 1 |
| SlowMo^8,9^ | SlowMo aims to improve paranoia and reasoning through digitally supported CBT for psychosis. In this study, individual face-to-face sessions were combined with digital access to coping strategies and interactive features (e.g. games and personalized thought bubbles) to help patients build awareness of unhelpful fast thinking and assist them in taking time to slow down | RCT of SlowMo + TAU vs TAU | 24 weeks | 361 (SlowMo 181; TAU 180) | SSD psychosis | 1, 2, 3, 4 |
|  |  | *Additional analysis to explore the subjective service-user experience* |  |  |  |  |
| PEAR-004^10^ | A smartphone investigational DT app designed to deliver multimodal evidence-based neurobehavioral mechanisms of action, including cognitive restructuring, illness self-management training, and social skills training to improve symptom management and functional outcomes | RCT of PEAR-004 vs non-specific digital sham | 12 weeks | 110 (PEAR-004 55; sham 55) | SZ | 1, 2, 3, 4 |
| MA-CBSST (Mobile-assisted cognitive-behavioral social skills training)^11^ | Cognitive behavioral social skills training was delivered through group therapy sessions. Handheld computers were used to supplement the sessions by delivering text-based prompts to support module-specific homework completion and by collecting brief self-monitoring ratings (on mood, voices, current activities, and medication adherence) | RCT with 3 arms: (1) 100% training time cognitive-behavioral social skills training (CBSST full protocol); (2) mobile-assisted CBSST (MA-CBSST) + 50% training time; (3) device contact (DC) for symptom monitoring only | 12 months | 57 (CBSST 26; MA-CBSST 17; DC only 14) | SZ, SZA | 1, 2, 4 |
| EMPOWER (Early signs Monitoring to Prevent relapse in psychosis and prOmote Wellbeing,  Engagement, and Recovery)^12-14^ | A smartphone app for self-monitoring blended with peer support and clinical triage to help individuals monitor wellbeing and detect early warning signs of relapse in schizophrenia, using a cognitive interpersonal model. | RCT of EMPOWER vs control (TAU) | 12 months | 73 (intervention 42; control 31) | SZ or related diagnosis | 1, 2, 3, 4 |
|  |  | *Additional analysis 1: Health Technology Assessment* |  |  |  |  |
|  |  | *Additional analysis 2: To explore implementation, including barriers and facilitators, from the point of view of carers, patients and mental health staff using qualitative interview* |  |  |  |  |
| MCI-S (Metacognitive intervention for schizophrenia)^15^ | Smartphone app with a metacognitive intervention program, which includes examples of psychotic symptoms to facilitate effective understanding and application of metacognition.  MCI-S app use was combined with mentoring sessions to provide patients with guidance on how to interpret and manage their psychotic symptoms and social contexts from the metacognitive perspective | Interventional non-equivalent comparative study with Experimental group (severe psychotic symptoms, low social functioning) who received MCI-S mobile app + weekly mentoring sessions, vs Comparison group (light psychotic symptoms, good social functioning who received app alone) | 10 weeks | 44 (severe symptom group 20; relatively light symptom group 24) | SZ | 2, 4 |
| SMARTapp (Schizophrenia Mobile Assessment and Real-Time feedback app)^16^ | Interactive smartphone app that aims to  improve daily life, social functioning, and symptoms using experience sampling method (ESM) feedback. Includes daily reminders (e.g., for medication and personal hygiene) and questionnaires to assess symptoms, social activities, mood, and thoughts about the day. Personalized interactive feedback provided (for patients in the feedback arm of the study) | RCT of SMARTapp + personalized feedback vs SMARTapp, no personalized feedback | 3 weeks | 50 (feedback 27; non-feedback 23) | SSD | 1, 2, 4 |
| ClinTouch^17^ | Personalized smartphone app for self-reporting of symptom severity in real time. Allowed detection of early signs of relapse by a clinical team | RCT of ClinTouch + TAU vs TAU | 12 weeks | 81 (intervention 40; control 41) | SZ and related disorders | 1, 2, 3, 4 |
| MEMS (Mobile Enhancement of Motivation in Schizophrenia)^18^ | Mobile text-messaging intervention, which aims to target impaired effort-cost computations and reduced future reward-value representation maintenance to improve patient motivation | RCT of MEMS + goal setting vs goal setting (control) | 8 weeks | 54 (intervention 25; control 29) | SSD | 1, 2, 4 |
| Florence^19^ | Interactive text-messaging system that includes medication and appointment reminders, daily individual wellbeing scores, and an option for additional patient support | RCT of Florence + TAU vs TAU | 6 months | 58 (intervention 29; control 29) | SZ, SZA, BD | 1, 2, 3, 4 |
| PRIME (Personalized real-time intervention for motivational enhancement)^20^ | A mobile app that provides text-based motivational coaching from trained therapists, personalized psychosocial goal setting, and peer-to-peer messaging. The app also includes a community feed to capture and reinforce rewarding experiences and progress towards goals | Stage 1: feasibility and acceptability assessment; Stage 2: Start of RCT of PRIME vs wait list / TAU (only results from first 10 participants reported) | 12 weeks + 12‑week follow-up | Stage 1: 10 Stage 2: results from first 10 participants reported | SSD | 1 |
| PRIME (Personalized real-time intervention for motivation enhancement)^21^ |  | RCT of PRIME vs  Waitlist + TAU (control) | 12 weeks | 43 (PRIME 22; control 21) | SSD | 1, 2, 4 |
| Digital shared decision-making smartphone app^22^ | Smartphone app to facilitate shared decision-making between patient and healthcare provider. Functions include preparation for consultation, daily self-assessments, action plans, and educational material. The app is synchronized to a web portal, enabling the patient’s healthcare provider to access patient responses prior to consultation | RCT of smartphone app + TAU vs TAU (control) | 6 months | 188 (intervention 90; control 98) | SZ, schizotypal or delusional disorder | 1, 2, 4 |
| Internet-based CBT for psychosis (iCBTp)^23,24^ | An internet-based self-help platform with optional smartphone app, for symptom-oriented, guided CBT as an intervention for psychosis | RCT of iCBTp vs wait list (WL; control group) with TAU for 8-wk RCT period, WL group subsequently given access to iCBTp | RCT, 8 weeks.  Follow-up assessment, 6 months | 101 (intervention 50; WL 51) | SSD | 1, 2, 3, 4 |
|  |  | *Secondary analysis to identify predictors of adherence and treatment outcomes* |  |  |  |  |
| **Non-comparative studies** | | | | | | |
| FOCUS^25,26^ | Smartphone app with pre-scheduled and on-demand interventions for real-time/real-place illness management support. Designed to improve coping with auditory hallucinations, social functioning, mood regulation, sleep and medication adherence. User data is uploaded to a dashboard that can be accessed by Health Technology Program case workers for relapse prevention planning. Developed to be used in conjunction with ongoing treatment | Interventional non-comparative study | 6 months | 342 | SSD | 1 |
|  |  | *Additional analysis to understand the implications of 24-hour/day, 7-day/week access to DTs* |  |  |  |  |
| FOCUS^27^ |  | Interventional non-comparative study | 1 month | 33 | SZ, SZA | 1, 2, 4 |
| FOCUS-AV (FOCUS-Audio/Video)^28^ | An adapted version of the FOCUS smartphone app (as above) with audio and video options | Interventional non-comparative study | 1 month | 9 | SZ, SZA | 1 |
| Self-management mobile app (unbranded)^29^ | Smartphone app designed to record and remind users to take their medication and participate in regular activities, such as eating breakfast, going food shopping, and cleaning their home. Physicians were able to tailor activity lists and review patients’ self-reported completions via a secure web interface | Interventional non-comparative study | 1 month | 14 | SZ | 1, 2, 4 |
| weCOPE^30,31^ | Smartphone app for illness self-management that includes 4 modules on symptom monitoring, problem-solving, anxiety management, and goal setting, and allows patients to contact their therapist in times of crisis | Interventional non-comparative study | 8 weeks | 9 | SZ | 2, 4 |
|  |  | *Additional analysis of the app development process* |  |  |  |  |
| ExPRESS (Experiences of Psychosis Relapse: Early Subjective Signs)^32,33^ | Smartphone app developed to monitor and assess early signs of relapse, and mood, basic, and psychotic symptoms | Interventional non-comparative study | 6 months | 18 | SSD | 1 |
|  |  | *Additional analysis on long-term acceptability* |  |  |  |  |
| MASS (Motivation and Skills Support)^34^ | Smartphone app designed to address social skills and motivation deficits in schizophrenia. The app is tailored to specific social goals and provides videos of actors demonstrating social skills to support patient goal attainment in daily life.  The app provides reminders and information for goal planning and conducts EMAs to evaluate affect and motivation in response to working towards social goals, and anticipated progress towards achieving goals | Interventional non-comparative study | 14 days | 8 | SZ, SZA | 1 |
| MASS (Motivation and Skills Support)^35,36^ |  | Interventional non-comparative study | 8-weeks with 3-month follow-up | 31 | SZ, SZA | 1, 2, 4 |
|  |  | *Additional analysis to examine the extent to which cognitive functioning is related to treatment engagement and outcome* |  |  |  |  |
| CBT2go^37^ | Smartphone app designed to prompt and track goal-directed activities in the community, facilitate  adherence to homework involving community  practice of thought-challenging skills, and prompt performance and savoring of planned, personalized pleasurable activities and social interactions. The app also used personalized statements to challenge social disinterest and defeatist attitudes in real-time, real-world environments. Blended with weekly in-person group sessions of CBT to modify defeatist attitudes and improve experiential negative symptoms | Interventional non-comparative study | 24 weeks | 31 | SZ, SZA | 1, 2, 4 |
| MATS (Mobile Assessment and Treatment for Schizophrenia)^38^ | Interactive mobile phone text-messaging that employs ambulatory monitoring methods and  CBT interventions. Developed to target medication adherence, socialization, and auditory hallucinations | Interventional non-comparative study | 12 weeks | 55 | SZ, SZA | 1, 2, 4 |
| m-RESIST (Mobile Therapeutic Attention for Patients with Treatment Resistant Schizophrenia)^39^ | An integrated mobile therapeutic intervention of psycho-education, clinical and psychological assessment, monitoring, therapeutic intervention and self-management for treatment-resistant schizophrenia. It comprises a wearable device (smartwatch) that captured GPS data, step counts, sleep patterns and heart rate, the m-RESIST app, a web-based platform and a tailored therapeutic program that recognizes early warning signs of psychosis to improve positive symptoms, treatment adherence, and healthy  lifestyle | Prospective non-comparative feasibility study | 3 months | 39 | SZ | 1, 3 |
| m-RESIST (Mobile Therapeutic Attention for Patients with Treatment Resistant Schizophrenia)^40^ |  | Qualitative, non-comparative study of  m-RESIST | Qualitative discussion | Patients’ group: 14 | SZ | 1 |
| A4i (App4Independence)^41^ | Smartphone app with real-time features (e.g., newsfeed) and offline features (e.g., toolkit and voice detector). A web-based provider portal facilitates set-up. The app addresses social isolation with personalized prompts, activity scheduling, and social engagement resources. It fosters recovery by providing evidence-based content tailored to individual needs related to symptoms. It includes a peer-peer engagement platform, daily wellness and goal attainment check-ins to guide content delivery and track mental health progress, and passively collects phone usage data as indicators of sleep and activity levels  In the descriptive A4i study, clinicians were connected with the provider portal and could view patients’ self-reported monitoring data (e.g. mood, sleep, adherence to medication). Clinicians also had access to dynamic risk flagging and indicators of patient wellness via the portal | Interventional non-comparative study | 1 month | 38 | SSD/other primary psychotic disorder | 1, 2, 3, 4 |
| A4i (App4Independence)^42^ |  | Descriptive study of A4i in 3 settings: *Context 1*: 2-year RCT + ad hoc A4i support and monitoring of engagement. *Context 2:* 16-month feasibility pilot with digital navigator support, monitoring of engagement, ad hoc support and education. *Context 3*: 6-month pilot facilitated by peer support workers) | Context 1: 2 years, Context 2: 16 months, Context 3: 6 months. All assessed over a 30- and 90-day period | Context 1: 58; Context 2: 9; Context 3: 116 | SSD | 1 |
| HYM (Heal Your Mind)^43^ | Smartphone app designed to assist individuals with early psychosis by providing real-time case management and self-directed CBT. Includes interactive functioning and modules on recording thoughts, symptoms, and daily life events. Official notices and communication modules serve as a group communication function and a scales module includes self-rating psychiatric scales | Interventional non-comparative study | One-time survey | 24 | Psychotic disorder | 1, 3 |
| MedActive ()^44^ | Interactive smartphone app and web-based clinician interface aimed at improving adherence to antipsychotic treatment. Daily EMAs keep track of medication adherence, positive psychotic symptoms, and medication side effects. Functions include automated reminders for medication administration and tailored motivational feedback is provided to patients to encourage adherence | Interventional non-comparative study | 2 weeks | 7 | SSD | 1 |
| MACS (Mobile After-Care Support)^45^ | Smartphone app designed using CBT-based strategies for psychosis. Monitors symptoms and treatment adherence, and includes interventions to support adherence and the use of healthy coping skills | Interventional non-comparative study of MACS | 1 month | 10 | SSD | 1, 2, 4 |
| MindFrame^46^ | Smartphone app that provides access to resources for self-management of living with schizophrenia. The app is affiliated with a website to support collaboration with the patient’s healthcare provider. App resources include self-assessment to monitor health, psychoeducation, early warning signs awareness, medication management, and strategies to be healthy | Qualitative analysis | 1 year | 27 | SZ | 1 |
| CT-155 beta^47,48,a^ | Software that can be accessed on mobile devices; designed to treat experiential negative symptoms and for use as an adjunctive to standard of care treatment. Provides schizophrenia-specific psychoeducation and introduces core therapeutic skills^48,a^ | Interventional non-comparative study | 3 weeks | 49 | SZ | 1 |
| IMPACHS m-health solution^49^ | Smartphone app designed to facilitate the transfer of CBTp-interventions into patients’ daily lives and monitor wellbeing, symptoms, and behavior. The app includes a psychoeducational manual and interactive e-learning-modules for addressing symptoms, experiences and difficulties associated with psychosis, individualized action plans, triggers, and self-assessments. Can be linked to a monitoring web-portal for clinicians | Interventional non-comparative study | 6 months, with option of app use for further 6 months | 24 | Psychosis | 1 |

BD, bipolar disorder; CBT, cognitive behavioral therapy; CBT-p, cognitive behavioral therapy for psychosis; DT, digital therapeutic; RCT, randomized controlled trial; SSD, schizophrenia spectrum disorder; SZ, schizophrenia; SZA, schizoaffective disorder; TAU, treatment as usual; WL, wait list.
^a^Name and description includes information taken from a more recent publication to supplement details available in original source identified from the literature search.

#### **Suppl. Table 4.** Studies relevant to Research Question 1: *Do people with schizophrenia/SSD use DTs and are they capable of using them effectively?*

| **Short citation** | **Digital intervention** | **Outcomes specific to research question #1** | **Results summary** |
| --- | --- | --- | --- |
| Bell et al. *Schizophr Res* 2020^1^  Moore E, et al. *Internet Interv* 2020^2^ | SAVVy  (MovisensXS)  None (interviews conducted ≤2 weeks after intervention period, except for 1 participant who was interviewed 1 month after trial completion) | Feasibility: Ecological momentary assessment (EMA) questionnaire completion rate (completers defined  as having completed over 33% of the total number of EMA questionnaires); % of participants for whom EMA-based feedback summaries produced; % of EMI reminders viewed; trial uptake and attrition; fidelity to intervention protocol.  Acceptability: Credibility and Expectancy Questionnaire (CEQ); Working Alliance Inventory-Short Revised (WAI-SR). | - Feasibility: Average completion rate of day and evening EMA questionnaires: 72% & 74%. Scheduled EMI reminders viewed on average 2.5 times/d, and 1.5 times/d when user-initiated. - Acceptability: Good satisfaction with intervention. 100% of treatment group would recommend to others  - Average WAI-SR score (max. 5) was 4.33 (SD 0.55), suggesting positive working alliance with therapist  - Average CEQ scores: Credibility subscale (max. 9): 7.5 (SD 1.56) for perceived logic of therapy; 6.68 (SD 1.53) for perceived success, and 6.56 (SD 2.21) for confidence in recommending therapy to others.  - Qualitative analysis showed 4 key themes: (1) Therapy experience changed by digital technology; (2) Valuing face-to-face component; (3) Preference for different phases of the digital technology; (4) Not as bothered by voices. Participants perceived EMI technology as helping capture their experience more accurately and communicating this more effectively to the therapist. |
| Ben-Zeev et al. *JMIR Ment Health* 2016^26^ | FOCUS | Engagement outcomes: - mHealth use  - Response to prompts  - Days of participant-initiated on-demand use - Average daily on-demand use.  Association between demographics and engagement outcomes. | - Engagement: Mean (SD) mHealth use: 3.5 (1.9) days/week; mean (SD) response to prompts: 2.9 (2.0) days/week; mean (SD) on-demand use: 1.8 (1.4) days/week; mean (SD) daily on-demand use: 1.2 (1.8); mean (SD) % of weeks used: 82 (21) %; mean (SD) % of weeks responding to prompts: 72 (28) %; mean (SD) % of weeks using on-demand functions: 62 (28) %; Use of intervention for 3-6 months: 252/342 (73.6%). Overall engagement declined over time: an average of 3.9 uses in the first week to 1.9 uses in Week 24. - Some demographics significantly associated with engagement outcomes (e.g., age and days of on-demand use: 46–60 vs 18–29 years, 0.48 days more weekly, p<0.001; age and daily on-demand use: 46–60 vs 18–29 years, 1.78 uses more per day, p<0.001; gender and days of mHealth use, females vs males, 0.42 days more weekly, p<0.01). |
| Ben-Zeev​ et al. *Psychiatric Rehab J* 2018^28^ | FOCUS-AV | Quantitative data on participants’ objective use of the mHealth intervention, evaluated via an 11-item measure of intervention modality preference, and a 12-item measure examining usability, acceptability and satisfaction of FOCUS-AV video content. | - Usage: Participants responded to 66.8% of system-delivered prompts to engage with FOCUS-AV, and 52.0% of FOCUS-AV use was initiated by participants. On average, FOCUS/AV was used 5.9 days/week, 4.4 times a day. Intervention modality preference: Participants used video functions an average of 28.3 times. They chose video over written interventions 66.7% of the times they used on-demand functions but opted for written content 77.7% of the times they responded to pre-scheduled prompts.  - Acceptability: 100% of the group would be willing to play the video on the bus with headphones and at home alone.  - Satisfaction: 88.8% were satisfied with how easy it was to use the videos. |
| Ben-Zeev​ et al. *Schizophr Bull* 2014^27^ | FOCUS | Acceptability/usability measure (adapted from System Usability Scale; Post Study System Usability Questionnaire; Usefulness, Satisfaction, and Ease of Use Questionnaire; and Technology Assessment Model Measurement Scales). | - Feasibility: 32/33 (97%) participants used the system successfully. Trial completers used the system on 86.5% of days, with an average of 5.2 times/d. ~62% of use of FOCUS was participant-initiated; 38% of use was in response to automated prompts.  - Acceptability: Participants found FOCUS easy to use (87.5%), felt very confident using it (90.3%) and were satisfied with it (90.6%). |
| Cinemre et al. *Stud Health Tech Inform* 2022^29^ | Self-management mobile app (unbranded) | Mobile app usage patterns. | - Ongoing resistance to recording medication (average: 5 entries/month) - Number of recorded activities of participants was variable (22–388); most frequent entries were: ate 171 (9.0%), washed face 163 (8.6%), brushed teeth 140 (7.4%), had breakfast 114 (6.0%), made bed 104 (5.5%). Daily recorded activities decreased over time (e.g., starting from ~100 entries/day, decreasing to <20/day by end of month). |
| Depp  et al. *Schizophr Bull* 2019^6^ | CBT2go | App adherence. | - Mean adherence to app use aggregated across modules was similar between the CBT2go and self-monitoring (SM) conditions (CBT2go, 68.7%; SM, 66.2%, p=0.413). Within individual modules, rates of adherence were higher for the evening survey on medication adherence in the CBT2go condition (p=0.025), but there were no other differences. |
| Eisner​  et al. *Schizophr Res* 2019^32^  Eisner et al. *JMIR Mhealth Uhealth* 2019^33^ | ExPRESS | Feasibility of weekly monitoring (engagement, completion of app assessments & phone interviews) App use in relation to symptoms of depression (Hospital Anxiety and Depression Scale) and fear of relapse (Fear of Recurrence Scale). | - 65% of app assessments and 58% of telephone interviews were completed. - Percentage app completion significantly and inversely correlated with baseline depression (p=0.015) and fear of relapse (p=0.014). - Retention: 16 of 18 participants completed the 6-month interview.  - Some reported finding it more accessible than visits from a clinician, as assessments were more frequent, more anonymous, and did not require the individual to explain their feelings in their own words. Nevertheless, barriers to app use (e.g., unfamiliarity with smartphones) were also reported.  A posteriori themes that were derived from the qualitative research included connection and accessibility,  self-reflection, therapeutic value, response style, and barriers to app engagement. |
| Fulford et al. *J Behav Cogn Ther* 2020^34^ | MASS | Acceptability and feasibility of MASS app (usability testing), and monitoring of ecological momentary assessment (EMA) entries. | - The MASS app demonstrated evidence of acceptability and engagement with app content.  - Frequency of completion of EMA surveys varied across users, ranging from <1/day to all 3/day. - Participants reported low ratings of difficulty with using the program and understanding the questions, and moderate ratings of enjoyment and helpfulness of the app. |
| Fulford et al. *JMIR Mental Health* 2021^35^ | MASS | Ecological momentary assessment (EMA) reporting, use of social skills training (SST) video content and response to push notifications. | - Number of social interactions reported via EMA decreased across 8-wk period (p=0.03). Number of interactions reported also showed a small, negative association with number of missing surveys (p=0.09); decline in interactions may have reflected survey burden, or that those with fewer interactions were less likely to engage with app. - Participants viewed SST videos an average of 13.5 (SD 14.52) times and responded to 42.5% (51/120) of push notifications on average. |
| Gallinat​ et al. *Int J Environ Res Public Health* 2021^7^ | HEINS | User Satisfaction and Program Utilization. | - Overall, participants receiving HEINS + treatment as usual were very satisfied with the program, with 100% liking the idea of weekly monitoring via mobile phone. In all participants, 70.7% of monitoring assessments were completed. On average, participants completed 19.08 of a maximum 27 monitoring questionnaires, and 8 participants triggered 27 monitoring alerts (mean 3.38 per person; SD 2.50). |
| Garety et al. *JAMA Psychiatry* 2021^8^  Greenwood et al. *Psychol Psychother Theory Res Pract* 2022^9^ | SlowMo | Session attendance metrics. Fidelity (≤1 web app component missed per session); mean calculated across all attended sessions. Mobile app adherence operationalized as ≥1 home screen interaction after at least 3 therapy sessions.  Exploration of the subjective service-user experience of SlowMo therapy content and design and the experience of the blended therapy approach, including triangle of therapeutic alliance and experience of digital aspects of intervention. | - Of 181 participants in the SlowMo arm, 145 (80.1%) completed all 8 therapy sessions. Therapy fidelity was high; of the 168 individuals who attended at least 1 session, 159 (94.6%) met a priori criteria for web app delivery, and 100/140 (71.4%) met adherence criteria for mobile app use (at least 1 home screen interaction after a minimum of 3 therapy sessions).  - Approaches and challenges of technology (further qualitative description reported):  **-** Phone app was seen as a tool to aid connectedness in daily life.  - For some people the combined cognitive and sensory demands of the blended therapy approach were seen as stimulating and therapeutic, whilst for others these were seen at times as overwhelming.  - A variety of challenges and issues with technology were described including a lack of interest in technology, limitations of the app interface or due to needing a second phone. |
| Ghaemi et al. *JMIR Form Res* 2022^10^ | PEAR-004 | Participant retention. Engagement metrics for both groups: time using app, days when app active, total number of sessions, and number of sessions per day). For PEAR-004 group, additional metrics were collected: number of skills practiced, repeated and mastered.  Subject Satisfaction Survey. | - Of 112 randomized participants, 92 (82.1%) completed the study (48/112, 85.7% from PEAR-004; 44/112, 78.6% from sham group). - App engagement was good: the PEAR-004 group spent more time (mean [SD], 4.2 [3.4] h/d) using the app than the sham group (2.2 [4.6] h/d) (p<0.001). - No significant differences between groups in mean number of days using app, total number of sessions, or number of sessions/day (all p>0.10). - Responses on the satisfaction survey (n=49) were generally positive; overall satisfaction rated 6 or 7 out of 8 by 73% of participants. |
| Granholm et al. *J Behav Cogn Ther* 2020^11^ | MA-CBSST | Participation assessed by homework adherence (rated on 6-point Likert scale). | - Groups (MA-CBSST, CBSST, device contact only [DC]) did not differ significantly in dropout rates at Weeks 12, 24 and Mo 12.  - 60% of participants responded at least daily to homework prompts, on average for 6 month (>168 prompts; DC 79%, MA-CBSST 44%). However, homework adherence was marginally significantly lower in the MA-CBSST group vs the full CBSST protocol (p=0.056). |
| Granholm​ et al*. JMIR Mental Health* 2020^37^ | CBT2go | App metrics and participant attendance & retention. | - Feasibility: Participant retention was excellent (87% at 18 weeks). Mean (SD) number of responses to 84 action plan prompts was 18.7 (21.3) or 22% at Week 12 and was 32.3 (31.5) to 168 action plan prompts (19.2%) at Week 24 for participants who did not drop out of treatment by each assessment point; this indicates engagement in homework and skills practice >1 time/week. |
| Granholm et al. *Schizophr Bull* 2012^38^ | MATS | Intervention completion relating to:  - Outcome assessments for medication adherence, socialization and auditory hallucinations  - Independent Living Skills Survey (ILSS) and Positive and Negative Syndrome Scale (PANSS). | - For the 42 completers, average valid response rate for 216 outcome assessment questions over 12 weeks was 86%, and 86% of phones were returned undamaged.  - Non-completers (n=13) had lower self-reported living skills (ILSS) and more severe negative symptoms (PANSS negative) than completers. |
| Grasa et al. *JMIR Form Res* 2023^39^ | m-RESIST | Feasibility: willingness to participate, dropout rate, non-use and compliance. Acceptability and usability: technological (perceived utility and ease of use, habit) and individual (attitude, barriers, intent to use) - per adapted Technology Acceptance Model (TAM) and Living Labs or Ecosystem of Open Innovation. Satisfaction: Client Satisfaction Questionnaire (CSQ-8). | Feasibility - 52% of people identified as candidates were receptive to participation.  - Dropout rate, 18%; non-use rate 0%. - Compliance: rate of reading messages 49.5%, questionnaire completions, ~40%. Acceptability - Positive results on TAM scale; mean score ≥5 (out of 7) in 6/7 dimensions. - >80% found m-RESIST user-friendly and easy to use. Satisfaction - 78% rated quality of service as excellent/good; 84% would recommend m-RESIST. - 94% were very/mostly satisfied; 84% would use it again.  - Mean total score of 25/32 on CSQ-8 scale. |
| Gumley et al. *Lancet Psychiatry* 2022^13^  Gumley et al. *Health Tech Access* 2022^14^  Allan et al. *BMC Psychiatry* 2023^12^ | EMPOWER | Feasibility (proportion of eligible service users who consented to enter the study, remained in the study, and provided relapse outcomes data. Feasibility assumed if criterion of >33% adherence to daily monitoring met). Acceptability (Mobile Application Rating Scale user version, uMARS). Usability (duration of app usage per participant and number of participants completing daily monitoring questionnaires).  Patient experience of using and implementing EMPOWER in daily life, experience of intervention components and data sharing.  Mental health staff experience of supporting patients taking part in EMPOWER, experience of interacting with EMPOWER in clinical practice and data. | Feasibility - 86 individuals consented to enter the study; 42 were randomized to EMPOWER and 31 to the control group (treatment as usual).  - At 12 months, outcomes data were available for 32 (76%) EMPOWER participants and 30 (97%) control participants.  - Of those randomized to EMPOWER, 30 (71%) met the criterion of >33% adherence to daily monitoring that assumed feasibility. Acceptability - uMARs ratings (scale 1–5) suggested app was interesting to use (ranging from 3.52–3.93 over 3 follow-ups), easy to learn (4.12–4.17), content well written (4.04–4.13), and content credible (4.45–4.57). Overall rating for app was positive (4.06–4.31 over 3 follow-up periods). Usability - 41 participants had the app set up and 33 (81%) completed a 4-week baseline monitoring period. Mean (SD) app use in the EMPOWER group was 31.5 weeks (14.5); within this period, participants used the app for an average of 4.5 days a week.  - The intervention was well implemented and EMPOWER was typically perceived positively by the 16 patients, 6 mental health staff and 1 carer interviewed. However, some patients reported negative views and reported ideas for intervention improvement. Patients reported valuing that the app afforded them access to things like information or increased social contact from peer support workers that went above and beyond that offered in routine care. Patients seemed motivated to continue implementing EMPOWER in daily life when they perceived it was creating positive change to their wellbeing, but seemed less motivated if this did not occur. |
| Hanssen et al. *Psychiatric Res* 2020^16^ | SMARTapp | Feasibility and acceptability of SMARTapp Response rate for Experience Sampling Method (ESM) questionnaires. | - Findings indicated good feasibility, with high compliance to the SMARTapp, which was rated as user-friendly and easy to understand. - Response rate for ESM questionnaires was 64% and 84% for self-initiated evening questionnaires. Completion rates did not change over the study, at 63.5%, 61.3% and 66.7% for Weeks 1, 2 and 3, respectively. - In the feedback group, participants reported acting on ≥1 personalized feedback prompt on 49% of study days. |
| Huerta-Ramos et al. *Actas Espanolas de Psiquiatria* 2017^40^ | m-RESIST | User needs and acceptability of m-RESIST, assessed from focus group discussion sessions and individual interviews. | - A webpage and a virtual forum were perceived as suitable to get reliable information on both the disease and support. Data transmission service, online visits, and instant messages were evaluated as ways to improve contact with clinicians. Alerts were appreciated as reminders of daily tasks and medical appointments. Positive acceptance of m-RESIST services was related to a perceived usefulness in meeting participants' needs and the possibility of maintaining human contact. |
| Kidd et al. *PLoS ONE* 2019^41^ | A4i | Feasibility. Engagement in recovery process (Personal Recovery Outcome Measure [PROM]). Usage of intervention (mHealth use and utility scale). | - A4i is feasible in terms of outcome and process indicators and is ready to progress to clinical trial and validation testing. - Engagement in recovery process: PROM scores showed improvement in recovery engagement but after correcting for comparisons, no significance was observed. No significant correlation between baseline (non-A4i) mobile phone use and change in PROM scores.  - Usage of intervention: Mean retention rate (% of total app users that return to the app on a specific day) was 52.5% (SD 22.13). Mean number of active interactions ranged from 6–654 in exposure period.  - Mean number of interactions per day was 4.21 (SD 5.19) and 68.3% of participants agreed they were satisfied with the app functions. |
| Kim et al. *Early Interv Psychiatry* 2018^43^ | HYM | Acceptance/usability survey. | - ~25% used the app at least once every 2 days; ~42% used it 1–2 times/week.  - Overuse or misuse of the app was not observed in participants, with no reports of addictive or poorly controlled use.  - ~83% found it easy to learn to use, 79% were satisfied with using it, and 71% received help as a result of using it. |
| Kreyenbuhl et al. *Clin Schizophr Relat Psychoses* 2019^44^ | MedActive | Feasibility and acceptability of app and clinician interface. | - Feasibility: All 7 participants completed the ~2 week trial (range: 14–18 days). They responded to 80% of all scheduled ecological momentary assessments and provided positive evaluations of app use. - Acceptability: 71% found using MedActive pleasant, 71% found the experience challenging, and 0% found it stressful. 100% agreed the app was easy to use and interesting; they also liked that their psychiatrist was able to view their medication adherence and how often they reported experiencing schizophrenia symptoms. Of note, 57% of participants needed technical support while using the intervention. |
| Lewis et al. *J Med Internet Res* 2020^17^ | ClinTouch | Feasibility and acceptability.  Percentage remaining in follow-up for 12 weeks completing >33% of all possible symptom ratings. | - Feasibility: 38/40 (95%) participants completed the 12-week trial; 84% of these 38 demonstrated acceptable adherence (responded to ≥33% of beep alerts) and 60% demonstrated good adherence (responded to >50% of beep alerts). |
| Luther et al. *J Consult Clin Psychol* 2020^18^ | MEMS | Intervention (MEMS) group assessments: Feasibility and engagement, assessed by examining text message response rates, and Usability, Satisfaction and Ease of Use questionnaire. | - Feasibility: Retention was high; 92.6% of 25 participants completed the 8-week study. - Engagement: Overall response rate to text messages in the MEMS group was high (86.1%). The higher text message response rate was significantly associated with greater improvement in effort-cost computations (p=0.001) and anticipatory pleasure (p=0.03). - Usability: 96% learned MEMS quickly and found it easy to use. 16% reported difficulties understanding text messages and typing responses and 12% reported difficulties operating their phone. - Satisfaction: 100% reported satisfaction with received text messages. 92% (n=23) reported the texts were useful, helped their motivation and helped them to reach their goals. |
| Moitra et al. *Psychiatric Quart* 2021^45^ | MACS | Feasibility and acceptability of the MACS app: - Study-designed phone usage questionnaire; catalogue of participants’ need for additional MACS training/trouble-shooting; Exit interview at 1-month follow-up; Client Satisfaction Questionnnaire-8 (CSQ-8); System Usability Scale (SUS); Usefulness, Satisfaction, and Ease of Use Questionnaire (USE). | - Feasibility: One participant (of 10) did not complete any MACS sessions. Total engagement (n=9) was reflected in ~1 session/day (average of 28); incomplete MACS sessions were rare (average of 7 sessions).  - Usability and acceptability: Overall positive satisfaction with MACS: mean rating of 2.4 on the 4-point CSQ-8 scale; positive SUS score (mean rating of 75.4, with scores >68 representing “above average” usability). Overall USE ratings in each subscale tended to the positive range. Participants engaged with a wide variety of MACS-provided coping skill interventions.  - Feedback in exit interviews was mixed but mainly positive. Most participants found the app easy to use. |
| Röhricht et al. *BMC Psychiatry* 2021^19^ | Florence | Intervention adherence: Recorded by measuring SMS response rates on the ‘Florence’ system. This involved recording daily well-being scores as well as the participant’s text inputs into the system. | - All participants received wellbeing reminders; none made use of the Request Support Function. Wellbeing scores were used by 27/29 participants allocated Florence; 20 used wellbeing monitoring consistently over the 6 months.  - 20 participants sent their wellbeing score at least on 50% of days (minimum of 15 per month) and mean number of wellbeing scores per month was 16.6. |
| Schlosser et al. *JMIR Res Protoc* 2016^20^ | PRIME | Acceptability: satisfaction ratings and trial retention. Feasibility: log-in frequency, average number of challenges completed, challenge completion percentage, average number of peer and coach interactions. | - Acceptability. Mean overall satisfaction at the 12-week assessment was 8 out of 10, with no significant difference between Stage 1 and 2 participants. (Stage 1 enrolled 10 participants to evaluate initial feasibility and acceptability; Stage 2 randomized participants to either PRIME or waitlist/treatment as usual only the results from the first 10 participants are reported here.) All participants were retained in the trial. - Feasibility: Participants logged into PRIME ~every other day; Stage 1: 3.51 times/week; Stage 2: 4.69 times/week. Participants were highly engaged in the platform (Stage 1: 177 direct messages sent to coaches; Stage 2: 955 messages to coaches (p=0.04). Participants from both stages completed an average of ~1.5 challenges/week and challenge completion percentage was high (>80%). |
| Schlosser et al. *Schizophr Bull* 2018^21^ | PRIME | Acceptability assessed at 12-week exit interview. Participants rated satisfaction with specific features (peer interaction, goal categories options) on a scale of 0, not at all, to 10, very much.  Feasibility was assessed by intervention metrics: login frequency, challenges completed, spontaneous and goal achievement moments, peer and coach interactions. | - Mean overall satisfaction with PRIME was 8.21 out of 10. - Most popular feature was ability to directly message coaches (mean 8.38); least popular was ability to track mood (6.33).  - Challenge completion rate was high (88%). - On average, participants logged in ~4 days/week. Over 12-weeks, participants were highly engaged in the platform, with 5152 direct messages sent from participants to coaches. In terms of peer-to-peer interactions, participants initiated interactions with each other a total of 497 times. |
| Sequeira et al. *Translat Behav Med* 2023^42^ | A4i | Number of engaged participants (used app ≥1 time in specified time period) in the first 30 and 90 days in each context. Retention rate and mean number of interactions. | - Study described implementation of A4i in 3 different clinical settings.  - Engagement with A4i was consistently high, as shown by number of participants engaged at 30 + 90 days:  Context 1: 30 days: 54/58; 90 days: 56/58  Context 2: 30 days: 9/9; 90 days: 9/9  Context 3: 30 days: 109/116; 90 days: 94/101  Total: 30 days: 172/183 (94%); 90 days: 159/168 (95%).  - Retention rate (%) at 30 and 90 days:  Context 1: 93/96  Context 2: 100/100  Context 3: 94/93.  - Mean number of app interactions at 30 and 90 days:  Context 1: 128/219  Context 2: 156/357  Context 3: 211/477. |
| Terp et al. *JMIR Ment Health* 2018^46^ | MindFrame | Qualitative evaluation of use and perspectives on app. | - Feasibility: Of the 77 individuals invited to use the app, 27 (35%) enrolled in the intervention period and agreed to use it; 13 participated in evaluation interviews. Evaluated participants described MindFrame as easy and intuitive to use. The period of MindFrame use was variable; 5 stopped using within 1 month, 4 stopped within 2–3 months, and 4 continued using for 6–12 months. Reasons for self-initiated termination included boredom, lack of motivation and energy, fatigue, and problems quantifying their mental health. |
| Vaidyanathan et al. *Am Psychiatry Assoc* 2023^47^ | CT-155 beta^48,a^ | Digital working alliance (DWA) assessed by mobile Agnew Relationship Measure (mARM). App engagement and lesson completion. | - mARM total and subscale scores showed a positive DWA was established after 1 week's use [median: 5.16, SD: 0.7] and maintained over the 3-week study [median: 5.18, SD: 0.8].  - mARM scores were positively correlated with overall engagement (number of sessions); p=0.01.  - Participants completed a median of 17 out of 18 lessons. |
| Vitger et al. *J Med Internet Res* 2022^22^ | Digital shared decision-making smartphone app | - Acceptance and perceived usefulness (4-item App Rating Questionnaire and 4-item Mobile App Rating Scale) - App use metrics | - 86/96 participants in the intervention group used the app and had a mean of 0.55 log-ins/day (equating to ~1 login every other day) during their active use period. The mean active use period was 39 days and participants saw an average of 20 different screens when using the app. - 55% (47/96) of participants in the intervention group logged in after the first month.  - Participants were somewhat satisfied with the app (mean score: 6.36 out of 12) and rated the app to be of average quality (mean score: 2.85 out of 5). There was no significant difference in treatment satisfaction between groups (as assessed using the client satisfaction questionnaire). |
| von Malachowski et al. *Schizophr Res* 2022^49^ | IMPACHS m-health solution | Solution (app) use over time. | Participants used the app for a mean of 2.91 days/week, more frequently in Month 1 (mean 4.25 days/week) than later months (Month 5: mean 2.10 days/week). Two participants did not use the app at all. A third of participants continued using the app after the 6-month study period. |
| Wester-mann et al.  *J Consult Clin Psychol* 2020^24^  Lüdtke et al. *Psychiatry Res* 2021^23^ | iCBTp | Web platform and smartphone app metrics, module completion data, and satisfaction with iCBTp intervention. | - Usage: 23% of the intervention group used the smartphone app. The web platform was used for a median (IQR) of 4 h 4 min (7 h 19 min), with a median of 12 (28.5) log-ins, 1 (3.5) message to a moderator, and the completion of 3 (6.0) modules and 5 (8.0) worksheets over the 8-week period. - Satisfaction with iCBTp intervention: Users were satisfied with the program’s quality (89%), type of help (66%), need orientation (66%), extent of help (68%), and practicality (75%). The majority would recommend the program to a friend (91%) or would use the program again (76%).  - In secondary analyses of data from the 8-week randomized controlled trial of iCBTp, regression analysis showed that none of the sociodemographic (e.g., age, gender, education), psychopathological (e.g., lack of insight, conviction of delusion, number of hospitalizations, dose of antipsychotic medication), or treatment-related variables (e.g., treatment credibility, working alliance) assessed were predictors of intervention adherence (defined as participant completion of at least 4 out of 8 modules); all p>0.05. |
| Ybarra et al*. J Nerv Mental Dis* 2022^5^ | T4RP | Feasibility: recruitment and retention rates  Acceptability:  - 12 questions for patients (5-point scale) covering intervention content, program experience, as well as intervention components posited to impact reductions in relapse risk  - 11 acceptability questions for providers (5-point scale)  Engagement: response to 80% of daily text messages in final week was deemed to support hypothesis of program engagement | - Feasibility: 88% completed 6-month follow-up, recruitment completed within 3 months, supporting hypothesis of feasibility.  - Acceptability: Over 90% of patients agreed or strongly agreed that the text messages were easy to understand, easy to answer, positive, and helped them feel supported. Over 90% of providers agreed or strongly agreed that the program made their patients feel supported and helped their patients better monitor their symptoms. Patient acceptability was positively associated with recovery and patient-provider communication scores; and negatively associated with symptoms of the disorder. 88% said they would recommend T4RP to other patients with schizophrenia.  - Program engagement: On average, participants responded to 80% of their program messages in the last week of the intervention, supporting hypothesis of program engagement. |

^a^Name includes information taken from a more recent publication to supplement details available in original source identified from the literature search.

#### Suppl. Table 5. Studies relevant to Research Question 2: *What is the efficacy/effectiveness of DTs in schizophrenia/SSD?*

| **Short citation** | **Digital intervention** | **Study design** | **Study duration** | **Population** | **Diagnosis** | **Outcomes specific to research question #2** | **Results summary** |
| --- | --- | --- | --- | --- | --- | --- | --- |
| Bell et al. *Schizophr Res* 2020^1^ | SAVVy (MovisensXS) | RCT of SAVVy (MovisensXS) + TAU vs TAU | 8 weeks | 34 (SAVVy + TAU 17; TAU 17) | SZ & other disorders | - Psychotic Symptom Rating  Scales-Auditory Hallucinations (PSYRATS-AH) total score - Depression, Anxiety and Stress Scale (DASS-21) overall score - Confidence in coping with voices day-to-day (visual analogue scales; VAS) - Awareness of patterns in voices (VAS) | - While there was a trend for improvement in PSYRATS-AH total score in favor of the intervention group, the difference between groups was not significant (p=0.09). - Small, non-significant improvement in DASS-21 scores in favor of control group (p=0.18). - Significant benefit in coping with voices in favor of intervention group (p<0.001). - Significant differences between groups in awareness of patterns in voices (p<0.05). |
| Ben-Zeev​ et al. *Schizophr Bull* 2014^27^ | FOCUS | Interventional non-comparative study | 1 month | 33 allocated | SZ, SZA | - Positive and Negative Syndrome Scale (PANSS)  - Beck Depression Inventory-2 (BDI-2)  - Insomnia Severity Index (ISI) | - Significant reductions in symptoms from pre-trial to post-trial on the PANSS total (p=0.002), PANSS positive (p<0.001), PANSS general psychopathology (p<0.001), and in depression on the BDI-2 (p=0.003). Scores on the PANSS-negative subscale did not significantly change.  No significant change in sleep difficulties (ISI). |
| Cinemre et al. *Stud Health Technol Inform* 2022^29^ | Self-management mobile app (unbranded) | Interventional non-comparative study | 1 month | 14 allocated | SZ | - Positive and Negative Syndrome Scale (PANSS) subscales: positive, negative, general psychopathology, total - Clinical Global Impressions Scale (CGI) | - Statistical significance after 1 month observed for PANSS positive signs, which improved (p=0.040). No significant improvement in PANSS negative, general psychopathology or total scores.  - CGI unchanged. |
| ClinicalTrials.gov NCT01969500^3^ | FOCUS | RCT of FOCUS vs TAU | Up to 12 weeks | 51 randomized (intervention 26; TAU 25) | SZ, SZA | - Change in Severity of Psychotic Symptoms: Psychotic Symptom Rating Scales (PSYRATS) | - No significant difference (p=0.85) between groups for change in severity of psychotic symptoms. |
| Cullen et al. *Psychiatry Res* 2020^4^ | T4RP | RCT of T4RP vs TAU | 6 months | 42 randomized (2:1 ratio intervention: control) | SZ, SZA | - Positive and Negative Syndrome Scale (PANSS) subscales: positive, negative, general psychopathology, total  - Montgomery-Asberg Depression Rating Scale (MADRS)  - Young Mania Rating Scale (YMRS)  - Recovery Assessment Scale Revised (RAS-R) - Brief Adherence Rating Scale (BARS), depot injection, and oral medication adherence rates  - Patient empowerment: Boston University Empowerment Scale (BUES) | - Positive PANSS scores significantly lower at 6-month follow-up (p=0.03) for intervention group.  - PANSS negative, general, and total scores were not significantly different between groups. - Symptoms of depression and mania not significantly different between groups. - Several indicators of recovery were significantly better for those in the intervention group at 3-month follow-up but significantly higher scores did not persist through to 6 months. - Improved oral medication adherence indicated at 3 and 6 months in intervention group (69% vs 89%, p=0.11). Greater rates of adherence to injectable medications were noted in the intervention group at 6 months (31% vs 100%, p=0.02).  - At 6 months, patient empowerment scores (BUES) were higher in the T4RP vs control group over time but not significantly different (p=0.15) |
| de Almeida et al*. J Technol Hum Serv* 2018^31^ | weCOPE | Interventional non-comparative study | 8 weeks | 9 allocated | SZ | - Recovery Assessment Scale (RAS) total and subscales - Positive and Negative Syndrome Scale (PANSS) positive, negative, and general psychopathology  - Empowerment Scale | - RAS total score (p=0.008) and subscales of for personal confidence and hope (p=0.011), goals and success orientation (p=0.033) and life beyond symptoms (p=0.033) all improved significantly.  - RAS willingness to ask for help (p=0.065) and confidence in others (p=0.168) not significant. - PANSS total score change not significant (p=0.062); PANSS positive score change not significant (p=0.180); PANSS negative score change not significant (p=0.655); general psychopathology subscale improved significantly (p=0.027).  - Empowerment Scale total score, and scores for self-esteem and confidence, optimism, and wrath were significantly different pre- and post-assessment (p<0.05). Empowerment Scale current power and community activism were not significantly different. |
| Depp et al. *Schizophr Bull* 2019^6^ | CBT2go | RCT with 3 arms: CBT2go; self-monitoring (SM); TAU | 24 weeks | 255 randomized (CBT2go 85, SM 85, TAU 85) | SZ, SZA, or BD | - Brief Psychiatric Rating Scale, BPRS-24 | - BPRS: Significant time × visit interaction for the 2 digital interventions vs TAU. No significant time × group interactions for CBT2go vs SM, or either intervention vs TAU. Treatment effects were small (d=0.23 at 2 weeks for CBT2go and d=0.22 for SM). - Average estimated improvement was significant for both digital interventions. |
| Fulford et al. *JMIR Ment Health* 2021^35^ | MASS | Interventional non-comparative study | 8-week intervention, 3-month follow-up | 37 allocated | SZ, SZA | - Brief Psychiatric Rating (BRP) Scale  - Clinical Assessment Interview for Negative Symptoms (CAINS) motivation and pleasure (MAP) | - BRP scale positive symptoms decreased significantly across the 3 time points.  - No significant changes in negative symptoms (CAINS-MAP scores) over time (baseline to follow up, p=0.83; baseline to termination, p=0.52). |
| Garety et al. *JAMA Psychiatry* 2021^8^ | SlowMo | RCT of SlowMo + treatment as usual (TAU) vs TAU | 24 weeks | 362 randomized (181 to SlowMo + TAU, 181 to TAU) | SSD, psychosis | - Self-reported paranoia severity: Green et al. Paranoid Thoughts Scale (GPTS) total score, GPTS Part A and B score - Secondary paranoia measures: Revised GPTS (R-GPTS) (total and subscale scores); Psychotic Symptom Rating Scales (PSYRATS) delusions subscale (total and 2 factors - conviction and distress); Scale for the Assessment of Positive Symptoms (SAPS), individual persecutory delusions and ideas and delusions of reference items  - Penn State Worry Questionnaire (PSWQ) | - No difference between groups in GPTS total score at 24 weeks (p=0.06).  - Significantly greater reductions with SlowMo on secondary paranoia outcomes at 24 weeks, including Part B score (p=0.04) but not Part A score (p=0.18), R-GPTS total score (P=0.03) and R-GPTS persecution score (p=0.04) but not R-GPTS social reference score (p=0.099). - Improvements were seen in PSYRATS total score (p=0.001), distress score (p=0.009), conviction score (p=0.001) at 24 weeks and in SAPS persecutory delusions score (P=0.009) and ideas and delusions of reference score (p=0.03).  PSWQ: At 24 weeks, mean (SD) scores for the SlowMo vs control group were 52.2 (11.6) and 54.5 (11.5), respectively; p=0.01. |
| Ghaemi et al.  *JMIR Form Res* 2022^10^ | PEAR-004 | RCT of PEAR-004 + TAU vs sham (control) + TAU | 12 weeks | 112 randomized (56 to PEAR-004, 56 to sham) | SZ | - Change in total Positive and Negative Syndrome Scale (PANSS) Score - PANSS subscales (general psychopathology, positive, and negative items)  - Motivation and Pleasure Self-report (MAP-SR) Score  - Beck Depression Inventory-2 (BDI-2) | - In the primary efficacy end point of change in total PANSS score from baseline to Day 85 or the last visit no benefit was seen with PEAR-004 vs sham. Small nondifferential improvement over time in both groups. At Day 85, the treatment mean difference between PEAR-004 and the sham group was 2.7 points in favor of the sham (2-sided p=0.09; 90% CI: 0.1, 5.4). - No notable benefits seen in secondary outcomes (PANSS subscales, total MAP-SR score, BDI-2) except for a small benefit for PEAR-004 in the BDI-2 total score at day 57 (least squares mean difference of 3.3 points, PEAR-004 vs sham); however, this difference did not persist at Day 85. |
| Granholm et al. *J Behav Cog Ther* 2020^11^ | MA-CBSST | RCT with 3 arms:  1. 100% training time cognitive-behavioral social skills training (CBSST full protocol);  2. Mobile-assisted CBSST (MA-CBSST) + 50% training time;  3. Device contact for symptom monitoring only (DC) | 12 months | 57 randomized (CBSST full protocol 26, MA-CBSST 17, DC 14) | SZ, SZA | - Clinical Symptoms: Positive and Negative Syndrome Scale (PANSS) and Scale for the Assessment of Negative Symptoms (SANS) (diminished expression and diminished motivation) | - No significant effects were seen for PANSS positive (p=0.715), SANS diminished motivation (p=0.361), SANS diminished expression (p=0.396). |
| Granholm​ et al. *JMIR Mental Health* 2020^37^ | CBT2go | Interventional non-comparative study | 24 weeks | 31 allocated | SZ, SZA | - Dysfunctional attitudes: Defeatist Performance Attitude Scale (DPAS) and Asocial Beliefs Scale (ABS) - Negative symptoms: Clinical Assessment Interview for Negative Symptoms Motivation and Pleasure subscale (CAINS-MAP); Clinical Assessment Interview for Negative Symptoms Expression subscale (CAINS-EXP) - Symptoms: Calgary Depression Scale (CDS), Positive and Negative Syndrome Scale (PANSS) positive subscale | Dysfunctional attitudes - Effect of time was significant for DPAS (γ=–0.59, t30=–4.27, p<0.001). Change in DPAS score from baseline was significant at all assessment points during treatment, with medium to large effect sizes. - Effect of time was not significant ABS. Symptoms - Significant reduction in severity of experiential negative symptoms (CAINS-MAP: γ=–0.14, t30=–3.12, p=0.004) with medium to large effect sizes. - Effect of time was not statistically significant for expressive negative symptoms/no significant reduction from baseline at any timepoint (CAINS-EXP).  - Significant reduction in severity of positive symptoms (PANSS) and depressive symptoms (CDS) was found by Week 24, but not at earlier assessment points. - Effect of time was not statistically significant for positive symptoms (PANSS) and depressive symptoms (CDS). |
| Granholm et al. *Schizophr Bull* 2012^38^ | MATS | Interventional non-comparative study | 12 weeks | 55 allocated | SZ, SZA | - Ambulatory monitoring of medication adherence and auditory hallucinations  - Positive and Negative Syndrome Scale (PANSS)  - Beck Depression Inventory - 2nd Edition (BDI-2) | - Medication adherence improved significantly in participants who were living independently.  - Significant reduction in severity of hallucinations on ambulatory monitoring. - Lab-based assessments of more general symptoms (PANSS and BDI-2) did not change significantly. |
| Gumley et al. *Lancet Psychiatry 2022^13^* | EMPOWER | RCT of EMPOWER vs TAU | 12 months | 73 randomized (intervention 42, control 31) | SZ or related diagnosis | - Change in Positive and Negative Syndrome Scale (PANSS) positive, negative, disorganization, excitement, emotional distress, and total score  - Hospital Anxiety and Depression Scale (HADS)  - Medication Adherence Rate Scale (MARS)  - PAM - anxiety  - Calgary Depression Scale (CDS) total score | - Negative PANSS domains showed significant change (p=0.004) over 12 months, no significant change in other PANSS domains or PANSS total score.  - Significant change in HADS depression subscale (p=0.010). No significant change in HADS anxiety subscale, PAM anxiety or CDS.  - Significant change in MARS over 12 months (p=0.047) (all exploratory analyses).  - No significant change in total CDS score (p=0.152). |
| Han et al. *J Psych-osoc Nurs Ment Health Serv* 2023^15^ | MCI-S | Interventional non-equivalent comparative study with Experimental group (severe psychotic symptoms, low social  functioning) who received MCI-S mobile app + weekly mentoring sessions, vs Comparison group (light psychotic symptoms, good social functioning who received app alone | 10 weeks | 50 allocated (24 severe symptom group, 26 relatively light symptom group) | SZ | - Change in psychotic symptoms (i.e., hallucinations and delusions) measured by the Psychotic Symptom Rating Scales (PSYRATS) - Change in Positive and Negative Syndrome Scale (PANSS) positive, negative, general psychopathology, and total | - In terms of Delusions, the experimental group showed significant differences in terms of change over time (p<0.001).  - There were no significant changes in either group for auditory hallucinations.  - PANSS total score in the experimental group significantly changed over time (p=0.010), but not for the comparison group. For the Negative Scale, the experimental group showed a significant change over time (p=0.002) compared to the comparison group, and for General Psychopathology, the experimental group significantly changed over time (p=0.027) compared to the comparison group. There were no significant changes in either group in for PANSS positive. |
| Hanssen et al. *Psychiatry Res* 2020^16^ | SMARTapp | RCT of SMARTapp + personalized feedback vs SMARTapp, no personalized feedback | 21 days | 64 randomized | SSD | - Community Assessment of Psychic Experiences (CAPE), positive, negative, depressive | - Questionnaire-assessed positive psychotic symptoms (CAPE measure) decreased significantly over time in both groups (p<0.01); no significant change in CAPE negative and depressive symptoms. |
| Kidd et al. *PLoS ONE* 2019^41^ | A4i | Interventional non-comparative study | 1 month | 38 allocated | SSD or other primary psychotic disorder | - Psychiatric symptomatology per Brief Symptom Inventory (BSI):  BSI - psychoticism  BSI – depression  BSI – phobic anxiety  BSI – obsessive compulsive disorder (OCD)  BSI – paranoid ideation  BSI – interpersonal sensitivity  BSI – total  BSI – somatization  BSI – hostility  BSI – anxiety  - Brief Adherence Rating Scale (BARS) | - Significant improvement was observed in some psychiatric symptom domains with small-medium effects.  - After controlling for gender, age, and baseline symptomatology, improvements were significant (p<0.05) for paranoid ideation, psychoticism, depression, phobic anxiety, OCD, and interpersonal sensitivity.  - Total BSI score, somatization, hostility, and anxiety were not significantly improved. - Improvements in BARS did not remain significant after correcting for comparisons. |
| Lewis et al*. J Med Internet Res* 2020^17^ | ClinTouch | RCT of ClinTouch + TAU vs TAU | 12 weeks | 81 randomized (intervention 40, control 41) | SZ and related disorders | - Positive and Negative Syndrome Scale (PANSS) total, positive, negative, general - Calgary Depression Scale (CDS) total score  - Empowerment Rating Scale (ERS) | - Although a significant reduction in positive symptoms was seen after 12 weeks of ClinTouch-enhanced monitoring in the early psychosis subsample at 1 investigational site (p=0.016), no significant difference between groups and across sites was seen in PANSS total, positive, negative, general scores after 6 or 12 weeks. - No significant differences on CDS and no significant difference on the ERS between groups at 12 weeks (p>0.05). |
| Luther et al.  *J Consult Clin Psychol* 2020^18^ | MEMS | RCT of MEMS + goal setting vs goal setting (control) | 8 weeks | 56 randomized (intervention 27, control 29) | SSD | - Clinical Assessment Interview for Negative Symptoms (CAINS) motivation, anticipatory pleasure, past week pleasure, expressive symptoms  - Positive and Negative Syndrome Scale (PANSS) Positive and mood symptoms - Motivation and Pleasure Scale - Self-Report (MAP-SR) motivation  - Quality of Life Scale (QLS) motivation index and motivation item  - Neurocognition: Brief Neurocognitive Assessment (BNA) | - Significant medium-sized group effects for CAINS motivation; MEMS demonstrated greater motivation vs control (p=0.03). - CAINS anticipatory pleasure significantly higher for MEMS vs control (p=0.02), with a medium effect size. - Higher text message response rate was significantly associated with greater improvement in anticipatory pleasure (p=0.03).  - No significant group effects were found for the QLS-Motivation Index (p=0.14)  - Participants in the MEMS group reached a significantly greater percentage of overall goals than those in the control group (p<0.001), with a large effect size. - No significant group differences for CAINS expressive negative symptoms or past week pleasure, PANSS positive symptoms or mood symptoms, or BNA neurocognition (all p>0.61).  - No significant group effects for MAP-SR motivation (p=0.61). |
| Moitra et al. *Psychiatric Quart* 2021^45^ | MACS | Interventional non-comparative study of MACS | 1 month | 10 | SSD | - Brief Psychiatric Rating Scale-18-item (BPRS) - Brief Adherence Rating Scale (BARS) - Brief Coping Orientation to Problems Experienced (Brief COPE) – dysfunctional coping | - Statistically significant reduction in psychiatric symptoms (BPRS; p=0.002). - Reported minimal antipsychotic medication nonadherence (4.7%) and outpatient treatment nonadherence (3.3%).  - Statistically significant reduction in dysfunctional coping strategies (COPE; p=0.002). |
| Röhricht et al. *BMC Psychiatry* 2021^19^ | Florence | RCT of Florence + TAU vs TAU | 6 months | 65 randomized (36 intervention, 29 control) | SZ, SZA, psychotic disorder un-specified, delusional disorder, BD | - Medication Adherence Rate Scale (MARS) | - No significant difference between intervention and control groups in medication adherence (MARS). |
| Schlosser et al. *Schizophr Bull* 2018^21^ | PRIME | RCT of  PRIME vs  Waitlist (WL) + TAU (Control) | 12 weeks | 43 randomized (22 PRIME, 21 control) | SSD | - Motivated behavior: modified version of Trust Task (anticipated pleasure and effort expenditure items)  - Change in motivation: Motivation and Pleasure Scale – Self-Report (MAP-SR) - Beck Depression Inventory (BDI-2) - Positive and negative symptoms: PANSS | - Motivated behavior: Significant difference in anticipated pleasure (p=0.03) and effort expended to increase the likelihood of future social interactions with positive outcomes (p=0.04); the increase from baseline to 12 weeks was greater for PRIME vs WL for both items (p=0.02 and p=0.03, respectively).  - Significant difference for depression symptoms (p=0.01); greater improvements from baseline to 12 weeks for PRIME vs WL (p=0.03).  - No significant improvement on the MAP-SR (p=0.06). - No differences in changes in PANSS. |
| Vitger et al. *J Med Internet Res* 2022^22^ | Digital shared decision-making smartphone app | RCT of smartphone app + TAU vs TAU alone (control) | 6 months | 194 randomized (96 intervention, 98 control) | SZ, schizo-typical or delusional disorder | - Scale for the Assessment of Positive Symptoms (SAPS) psychotic and disorganized - Scale for the Assessment of Negative Symptoms (SANS)  - Adult State Hope Scale | - No statistically significant differences between the groups for SAPS psychotic (p=0.10) or disorganized (p=0.71) SANS (p=0.13) or Hope Scale (p=0.12). |
| Wester-mann et al. *J Consult Clin Psychol* 2020^24^ | iCBTp | RCT of iCBTp + TAU vs wait list (WL) + TAU (control group subsequently given access to iCBTp) | RCT: 8 wks. Follow-up assessment (inc. control receiving intervention after 8 weeks) at 6 months | 101 randomized (intervention 50, control 51) | SSD | - Launay–Slade Hallucination Scale (LSHS) - Paranoia Checklist (PC)  - PANSS – positive, negative, and general  - Penn State Worry Questionnaire (PSWQ)  - Patient Health Questionnaire (PHQ) | - Interaction was significant for the individual primary endpoint LSHS (hallucination severity) but not significant for PC (paranoia), PANSS positive, negative or general, PSWQ scores, or PHQ depression. - At 6-month follow-up, positive effects on paranoia, hallucinatory experiences, positive symptom severity increased further or remained stable. None of the outcomes significantly deteriorated during the follow-up period. |

####

#### Suppl. Table 6. Outcomes with no significant differences between groups (comparative studies) or between pre- and post-intervention (non-comparative studies) for Research Question 2: *What is the efficacy/effectiveness of DTs in schizophrenia/SSD?*

| **Comparative studies** | | | | | | | | |
| --- | --- | --- | --- | --- | --- | --- | --- | --- |
| **DT** | **Control** | **Outcomes with no significant between-group difference (DT vs control)** | | | | | | |
|  |  | **Negative symptoms** | **Positive symptoms** | **Cognitive performance** | **Mood, depression and/or anxiety** | **Medication adherence** | **Recovery** | **General/ other symptoms** |
| SAVVy + TAU^1^ | TAU |  | ✓ ^a^ |  | ✓ ^b^ |  |  |  |
| FOCUS^3^ | TAU |  | ✓ ^a^ |  |  |  |  |  |
| T4RP ^4^ | TAU | ✓ ^a^ |  |  | ✓ (2)^a^ | ✓ ^a^ | ✓ (7) ^b^ | ✓ (2) ^a^ |
| CBT2go^6^ | TAU |  |  |  |  |  |  | ✓ ^a^ |
| SlowMo + TAU^8^ | TAU |  | ✓ (3)^b^ |  |  |  |  |  |
| PEAR-004 + TAU^10^ | Digital sham + TAU | ✓ (2 [1^a^ + 1^b^]) | ✓ ^a^ |  | ✓^b^ |  |  | ✓ (2) ^a^ |
| MA-CBSST^11^ | Device contact only^c^ | ✓ (2) ^a^ | ✓^a^ |  |  |  |  |  |
| EMPOWER^13^ | TAU |  | ✓ ^a^ |  | ✓ (3 [2^a^ + 1^b^]) |  | ✓ ^a^ | ✓ (4) ^a^ |
| MCI-S mobile app + weekly monitoring^15^ | MCI-S mobile app |  | ✓ (2) ^a^ |  |  |  |  |  |
| SMARTapp + personalized feedback^16^ | SMARTapp, no personalized feedback | ✓ ^b^ |  |  | ✓ ^b^ |  |  |  |
| ClinTouch + TAU^17^ | TAU | ✓ ^a^ | ✓ ^a^ |  | ✓ ^a^ |  | ✓ ^a^ | ✓ (2) ^a^ |
| MEMS + goal setting^18^ | Goal setting | ✓ (4 [3^a^ + 1^b^]) | ✓ ^a^ | ✓ ^a^ | ✓ ^a^ |  |  |  |
| Florence + TAU^19^ | TAU |  |  |  |  | ✓ ^b^ |  |  |
| PRIME^21^ | WL + TAU | ✓ ^b^ |  |  |  |  |  | ✓ ^a^ |
| Smartphone app + TAU^22^ | TAU | ✓^a^ | ✓ ^a^ |  | ✓ ^b^ |  |  | ✓ ^a^ |
| iCBTp + TAU^24^ | WL + TAU | ✓ ^a^ | ✓ (2 [1^a^ + 1^b^]) |  | ✓ (2) ^b^ |  |  | ✓ (2 [1^a^ + 1^b^]) |
| **Non-comparative studies** | | | | | | | | |
| **DT** | | **Outcomes demonstrating no significant difference between pre- and post-DT intervention** | | | | | | |
|  |  | **Negative symptoms** | **Positive symptoms** | **Cognitive performance** | **Mood, depression and/or anxiety** | **Medication adherence** | **Recovery** | **General/ other symptoms** |
| FOCUS^27^ | | ✓ ^a^ |  |  |  |  |  | ✓ ^b^ |
| Self-management mobile app^29^ | | ✓ ^a^ |  |  |  |  |  | ✓ (3) ^a^ |
| weCOPE^31^ | | ✓ ^a^ | ✓ ^a^ |  |  |  | ✓ (4) ^b^ | ✓ ^a^ |
| MASS^35^ | | ✓ ^a^ |  |  |  |  |  |  |
| CBT2go^37^ | | ✓ ^a^ |  |  | ✓ ^a^ |  |  |  |
| MATS^38^ | |  |  |  | ✓ ^b^ |  |  | ✓^a^ |
| A4i^41^ | |  |  |  | ✓ ^b, d^ | ✓ ^a, e^ |  | ✓ (3) ^b, e^ |
| MACS^45^ | |  |  |  |  | ✓ ^a^ | ✓ (2) ^b^ |  |

^a^Clinician/researcher/interviewer assessed; ^b^self-reported; ^c^device contact and symptom monitoring without CBSST; ^d^after controlling for gender, age, and baseline symptomatology; ^e^after controlling for covariates. Where there is more than 1 outcome type in a table cell, the number is given in brackets.

Outcomes with significant differences between groups or between pre- and post-intervention for Research Question 2 are shown in **Table 1.**DT, digital therapeutic; TAU, treatment as usual. For full DT names, see Appendix at the end of this supplement.

#### Suppl. Table 7. Studies relevant to Research Question 3: *What are the safety concerns associated with DTs in schizophrenia/SSD?*

| **Short citation** | **Digital intervention** | **Study design** | **Study duration** | **Population** | **Diagnosis** | **Outcomes specific**  **to research question #3** | **Results summary** |
| --- | --- | --- | --- | --- | --- | --- | --- |
| Bell et al. *Schizophr Res* 2020^1^ | SAVVy (MovisensXS) | RCT of ecological momentary assessment/  intervention (EMA/I) intervention blended with 4 face-to-face sessions + treatment as usual (TAU) vs TAU | 8 weeks | 34 (SAVVy + TAU 17; TAU 17) | SZ & other disorders | Not stated | - 2 serious adverse events (SAEs) (hospital admissions) were reported; 1 in the intervention and 1 in the control group. Both were considered unrelated to trial or intervention. |
| ClinicalTrials.gov NCT01969500^3^ | FOCUS | RCT of FOCUS vs TAU | Up to 12 weeks | 51 (intervention 26; TAU 25) | SZ, SZA | Adverse events were monitored at baseline and 12 weeks | - 1 all-cause mortality event in intervention group; no other events reported |
| Depp et al. *Schizophr Bull* 2019^6^ | CBT2go | RCT with 3 arms: CBT2go; self-monitoring (SM); TAU | 24 weeks | 255 (CBT2go 85, SM 85, TAU 85) | SZ, SZA, BD | Adverse event (AE) data were collected. The study was monitored by a Data and Safety Monitoring Board (DSMB) annually. Timing, duration, and aftermath of psychiatric and nonpsychiatric hospitalizations were collected. | - 31 AEs were experienced by 21 different participants (SM, n=12; CBT2go, n=10, and TAU, n=9).  - All AEs were hospitalizations; 12 medical and 19 psychiatric. These AEs were reported to the DSMB and determined unlikely to be related to the study interventions and consistent population risk. |
| Garety et al. *JAMA Psychiatry* 2021^8^ | SlowMo | RCT of SlowMo + TAU vs TAU | 24 weeks | 362 randomized, 363 included in ITT analysis | SSD, psychosis | Adverse events (AEs) were monitored throughout the study until the 24-week follow-up. | - 54 AEs were reported, of which 51 were serious adverse events (SAEs) (25 SAEs occurred in 19 participants in the SlowMo group; 26 SAEs occurred in 21 participants in the TAU group).  - The relationship of serious AEs to trial participation, in the SlowMo and TAU groups, respectively, was (n [%]):  - Not related, 23 (92.0), 25 (96.2)  - Unlikely related, 1 (4.0), 0 (0)  - Possibly related, 1 (4.0), 0 (0)  - Definitely related, 0 (0), 1 (3.8); this 1 SAE definitely related to trial participation in the TAU (control) group involved a complaint when the research team shared information with the clinical team under a duty of care (confirmed by independent ethical review). The participant subsequently requested to withdraw data and was thus considered a ‘post-randomization exclusion’. - Common AEs in participants in the SlowMo and TAU groups, respectively included:  - Admission to psychiatric hospital during follow-up, n=8, n=10  - Referral to crisis care, n=5, n=2  - Physical AEs, n=8, n=2  - Other AEs, n=5, n=4 - No deaths were recorded. - Intensity of AEs in the SlowMo and TAU groups, reported as n (%), respectively, was: Mild, 2 (7.1), 0 (0); Moderate, 11 (39.3), 10 (38.5); Severe, 15 (53.6), 16 (61.5) |
| Ghaemi et al. *JMIR Form Res* 2022^10^ | PEAR-004 | RCT of PEAR-004 vs sham app | 12 weeks | 112 randomized (56 to PEAR-004, 56 to sham) | SZ | Safety assessments consisted of collecting all adverse events (AEs), serious adverse events (SAEs), vital signs and the InterSePT Scale for Suicidal Thinking–Plus (with a specific focus on Part 3 for assessment of severity of suicidal risk, summarized by treatment group and time visit). | - AEs were reported in 20% (22/110) of participants.  - Incidence of AEs was similar across groups: 22% (12/55) in the PEAR-004 group and 18% (10/55) in the sham group.  - All reported AEs were categorized as mild (20/110, 18.2%) or moderate (2/110, 1.8%) in severity.  - No severe AEs were reported.  - Most AEs were not suspected to be related to treatment and resolved or were recovering at end of study.  - One SAE (suicidal ideation) was reported in the sham group, and the participant was discontinued from the study. This event was considered resolved on Day 43 and was not considered related to treatment. - No clinically significant abnormalities related to vital signs were reported. |
| Grasa et al. *JMIR Form Res* 2023^39^ | m-RESIST | Prospective feasibility study, non-comparative | 3 months | 39 | SZ | Participants were monitored for incidence of adverse events | - Three serious adverse events (SAEs) were reported that required hospitalization due to worsening of psychotic symptoms.  - No direct association between these SAEs and the m-RESIST intervention or the protocol procedures was found.  - The most probable causes were the evolution of the clinical pattern in 2 participants and a decrease in the sleep pattern in the third participant. |
| Gumley et al. *Lancet Psychiatry 2022^13^* | EMPOWER | RCT of EMPOWER vs TAU | 12 months | 73 (intervention 42, control 31) | SZ or related diagnosis | Adverse events (AEs) monitored for safety. Participant responses on Fear of Recurrence Scale (FoRSe) | - In the EMPOWER group (n=42), 29 AEs affected 19 (45%) participants, with 11 events classified as serious adverse events (SAEs). One SAE in the EMPOWER group resulted in death, but was not considered related to use of the app. - In the TAU group (n=31), 25 AEs affecting 10 people (32%) with 15 events classified as SAEs. - Intensity of SAEs: EMPOWER: Mild (1/11; 9%), Moderate (1/11; 9%); severe (9/11; 82%) TAU: No mild or moderate SAEs. Severe, 15 (100%) - The study identified 13 app-related AEs that affected 11 people in the EMPOWER group, one of which was serious (hospital admission in part related to feeling overwhelmed at the point of app installation). No app-related AEs were reported in the TAU group. - Fear of relapse was lower in the EMPOWER group than in the TAU group at 12 months (mean difference –7.53 (95% CI: –14.45, 0.60, Cohen’s d –0.53), suggesting routine monitoring did not exacerbate hypervigilance or anxiety about illness. |
| Kidd et al. *PLoS ONE* 2019^41^ | A4i | Interventional non-comparative study | 1 month | 38 | SSD or other primary psychotic disorder | Not stated | - One participant reported anxiety regarding text messages which made them feel that someone was monitoring them but also fed back that, if A4i were to become available, they would definitely use it. Aside from this finding, no unintended or adverse events were observed related to A4i use. |
| Kim et al. *Early Interv Psychiatry* 2018^43^ | HYM | Interventional non-comparative study | One-time survey | 24 | Psychotic disorder | Not stated | - No adverse events related to the app were observed while participants were using it. - 95.8% of participants did not find their symptoms exacerbated by using the app. |
| Lewis et al. *J Med Internet Res* 2020^17^ | ClinTouch | RCT of ClinTouch + TAU vs TAU | 12 weeks | 81 (intervention 40, control 41) | SZ & related disorders | Safety was routinely monitored during weekly telephone support calls | - Of 38 participants who completed 12 weeks of the trial, 3 (8%) reported significant adverse events: 1 reported increased anxiety prompted by questions; 1 reported increased irritation due to the alert beeps, and 1 had their charger explode. All 3 participants continued to complete the 12 weeks of the trial. |
| Röhricht et al. *BMC Psychiatry* 2021^19^ | Florence | RCT of Florence + TAU vs TAU | 6 months | 65 randomized (36 intervention, 29 control), 56 follow-up, 56 analysis | SZ, SZA, psychotic disorder unspecified, delusional disorder, bipolar disorder | Not stated | - No harmful effects were observed as a result of the trial. |
| Wester-mann et al. *J Consult Clin Psychol* 2020^24^ | iCBTp | RCT of iCBTp + TAU vs wait list (WL; control group with TAU for 8-wk RCT period, subsequently given access to iCBTp) | RCT: 8 weeks Follow-up assessment (including wait-list control receiving intervention after 8 weeks): 6 months | 101 (Intervention 50, control 51) | SSD | Questionnaire about Side Effects Psychosis and Internet | - 11 participants experienced an adverse event (AE) during the intervention (11%; iCBTp: 3, WaitList: 8); 8 events were not related to the study (73%), and none definitely related to the study (0%). The remaining 3 AEs that potentially related to the study (27%) involved a change in anti-psychotic medication (2, both in WL) and the experience of a vision while filling in a questionnaire (1, in iCBTp). - The number of negative experiences/effects reported by the 80 participants who filled in the corresponding questionnaire (QueSPI) ranged from 0 to 15, and 21% of participants reported no negative experiences/effects. The most frequent negative experience/effect (38% of the 80 participants) was “Human contact was missing in the self-help program via the Internet”. |

#### Suppl. Table 8. Studies relevant to Research Question 4: *What is the impact of DTs in schizophrenia/SSD on real-world functioning, quality of life, and other outcomes?*

| **Short citation** | **Digital intervention** | **Study design** | **Study duration** | **Population** | **Diagnosis** | **Outcomes specific to research question #4** | **Results summary** |
| --- | --- | --- | --- | --- | --- | --- | --- |
| Bell et al. *Schizophr Res* 2020^1^ | SAVVy (MoviesensXS) | RCT of SAVVy (MovisensXS) + TAU vs TAU | 8 weeks | 34 (SAVVy + TAU 17; TAU 17) | SZ & other disorders | - Subjective Experiences of Psychosis Scale (SEPS) negative impact subscale total score | - Small, non-significant improvement in SEPS negative impact subscale in favor of intervention group (p=0.46). |
| Ben-Zeev​ et al. *Schizophr Bull* 2014^27^ | FOCUS | Interventional non-comparative study | 1 month | 33 allocated | SZ, SZA | - Brief Medication Questionnaire (BMQ) assessments | - No significant changes in beliefs about medications (BMQ general and BMQ necessity-concern differential scores). |
| Cinemre et al. *Stud Health Tech Inform* 2022^29^ | Self-management mobile app (unbranded) | Interventional non-comparative study | 1 month | 14 recruited, | SZ | - Instrumental Activities of Daily Living Scale (IADLS)  - Functional Remission of General Schizophrenia Scale (FROGS) social functioning, FROGS health and treatment, FROGS occupational functioning, FROGS total, FROGS daily life skills | - No significant change in IADLS score (pre- and post-study scores were identical; p=0.317), FROGS social functioning (p=0.809), FROGS health and treatment (0.586), FROGS occupational functioning (0.339) or FROGS total (p=0.069) scores.  - Statistical significance after 1 month observed for FROGS daily life skills, which deteriorated (p=0.012). |
| ClinicalTrials.gov NCT01969500^3^ | FOCUS | RCT of FOCUS vs TAU | Up to 12 weeks | 51 (intervention 26; TAU 25) | SZ, SZA | - Change in Social Functioning: 2 subscales from the Social Functioning Scale (SFS) | - No significant difference (p=0.44) between groups for change in social functioning. |
| Cullen  et al. *Psychiatry Res* 2020^4^ | T4RP | RCT of T4RP vs TAU | 6 months | 42 randomized (2:1 ratio of intervention: control) | SZ, SZA | - Institutionalization: rates of hospitalization, emergency room visits, intensive outpatient program referrals  - Improving communication between patients and providers | - No significant differences between groups for institutionalization (rates of hospitalization, emergency room visits, intensive outpatient program referrals). - No difference between groups in patient-provider communication ‘score’ at 6 months. |
| de Almeida et al.  *J Tech Human Services* 2018^31^ | weCOPE | Interventional non-comparative study | 8 weeks | 9 allocated | SZ | - Empowerment Scale (total score plus 5 subscales) & Social Support Satisfaction Scale (total score plus 4 domains), assessed pre- and post- 8 weeks of app use  - Personal and Social Performance Scale (PSPS)  - General self-efficacy scale (GSES) (total score plus 2 domains) | - Vs pre-app use, Empowerment Scale total scores were significantly different (p=0.017); by subscale, significant values (p<0.05) were seen for self-esteem & confidence, optimism and wrath (righteous anger), but not for current power or community activism.  - Social Support Satisfaction total scores were significantly different (p=0.021); by subscale, significant values were seen for intimacy and satisfaction with family (both p<0.05) but not for satisfaction with friends or social activities.  - PSPS total score improved significantly (p=0.012).  - GSES total score (p=0.007) and subscales of initiation and persistence (p=0.008) and effectiveness in adversity (p=0.011) all improved significantly but there was no significant difference in GSES social effectiveness. |
| Depp et al. *Schizophr Bull* 2019^6^ | CBT2go | RCT with 3 arms: CBT2go; self-monitoring; TAU | 24 weeks | 255 randomized (CBT2go 85, SM 85, TAU 85) | SZ, SZA, or BD | - Specific Level of Functioning Scale (SLOF)  - Defeatist Performance Attitude Scale (DPAS) | - SLOF results showed significant group × time effect for community functioning, favoring CBT2go vs TAU (p=0.046). Treatment effects were small–medium at 24 weeks for CBT2go (d=0.36).  - DPAS: No time × group interactions; however, estimated change indicated significant improvement in the CBT2go condition but not in the SM condition. |
| Fulford et al. *JMIR Mental Health* 2021^35^ | MASS | Interventional non-comparative study | 8-week intervention, 3-month follow-up | 31 | SZ, SZA | - Social engagement per Heinrichs Quality of Life Scale–Interpersonal Relations (QLS-IR) subscale  - Social functioning Scale (SFS) | - QLS-IR scores showed a small but non-significant increase from baseline to treatment termination, followed by a non-significant decrease at follow-up.  - Improved SFS scores from BL to treatment termination, particularly in females; however, gains not maintained at 3-month follow-up (mean SFS scores showed moderate increase from BL to treatment termination [p=0.02], but decreased at follow-up [p=0.09]). |
| Garety et al. *JAMA Psychiatry* 2021^8^ | SlowMo | RCT of SlowMo + TAU vs TAU | 24 weeks | 362 randomized, 363 included in ITT analysis | SSD psychosis | - Manchester Short Assessment of Quality of Life  - Warwick-Edinburgh Mental  Well-being Scale (WEMWBS)  - Brief Core Schema Scale (BCSS) negative and positive self and negative and positive other scores  - Reasoning: Fast and Slow Thinking Questionnaire | - Significant improvements at Week 24 were found for participants allocated to SlowMo in quality of life (p=0.003) and wellbeing (p=0.001).  BCSS negative self-score (p=0.01) and BCSS positive self-score (p=0.006) were also significantly improved at 24 weeks.  - There were no significant differences in BCSS negative other score (p=0.77) or BCSS positive other score (p=0.42).  - The fast- and slow-thinking scale of the Fast and Slow Thinking Questionnaire showed improvements at 24 weeks (p=0.004 and p=0.03, respectively). |
| Ghaemi et al.  *JMIR Form Res* 2022^10^ | PEAR-004 | RCT of PEAR-004 + TAU vs sham (control) + TAU | 12 weeks | 112 randomized (56 to PEAR-004, 56 to sham) | SZ | - World Health Organization Quality of Life (WHOQOL)-BREF domains 1–4 | - No significant difference between groups in WHOQOL-BREF domains 1–4. |
| Granholm​ et al. *J Behav Cog Ther* 2020^11^ | MA-CBSST | RCT with 3 arms:  1. 100% training time cognitive-behavioral social skills training (CBSST full protocol);  2. Mobile-assisted CBSST (MA-CBSST) + 50% training time; 3. Device contact for symptom monitoring only (DC) | 12 months | 57 randomized (CBSST full protocol 26, MA-CBSST 17, DC 14) | SZ, SZA | - Independent Living Skills Survey (ILSS)  - Cognitive insight: BCIS  - Comprehensive Module Test (CMT)  - Maryland Assessment of Social Competence (MASC) effectiveness  - Defeatist Performance Attitudes Scale: DPAS | - While statistically significant CBSST group x time interactions were found for ILSS, indicating significantly greater improvements over time, they were not found for the mobile-assisted CBSST.  - No significant effects were seen for any outcome: CMT (0.056), MASC effectiveness (p=0.911), BCIS index (p=0.518), and DPAS (p=0.898). |
| Granholm et al. *Schizophr Bull* 2012^38^ | MATS | Interventional non-comparative study | 12 weeks | 55 (enrolled) 42 (active participants) | SZ, SZA | - Independent Living Skills Survey (ILSS)  - Ambulatory monitoring of socialization | - There were no significant differences between baseline and post-intervention assessment in ILSS scores.  - Significant increase in social interactions. |
| Granholm​ et al*. JMIR Mental Health* 2020^37^ | CBT2go | Interventional non-comparative study | 24 weeks | 31 | SZ, SZA | - Abbreviated Quality of Life Scale (A-QLS)  - Social Functioning Scale (SFS)  - Defeatist Performance Attitude Scale (DPAS)  - Asocial Beliefs Scale (ABS) | - A-QLS scores were significantly improved at 12 weeks but not at 24 weeks and the effect of time on A-QLS scores was not significant (p=0.15).  - Significant improvement on SFS total score between baseline and 12 weeks but not at Week 24; effect of time was only at a trend level (p=0.09).  Changes in DPAS scores over time were significant at Weeks 12 and 24 (both p<0.05).  There were no significant changes in mean scores on the ABS at any time point. |
| Gumley et al. *Lancet Psychiatry 2022^13^* | EMPOWER | RCT of EMPOWER vs TAU | 12 months | 73 (intervention 42, control 31) | SZ or related diagnosis | - Personal and Social Performance Scale (PSP)  - Personal Beliefs about Illness Questionnaire-Revised (PBIQ-R) – control, shame, entrapment, loss, social marginalization  - Participant engagement assessed by service provider (Service Engagement Scale, SES) – availability, collaboration, help seeking, and treatment adherence domains, and total SES score  - General self-efficacy scale (GSES)  - Patient activation measure (PAM) – avoidance  - Service attachment questionnaire (SAQ) | - Significant improvements in PBIQ-R control scores (p=0.045) . - No significant changes in the other PBIQI domains, PSP, GSES, PAM - avoidance or SAQ.  - Significant improvements were reported on the treatment adherence domain of the SES (p=0.016). No significant differences were seen on the remaining SES domains or for total SES scores. |
| Han et al. *J Psych-osoc Nurs Ment Health Serv* 2023^15^ | MCI-S | Interventional non-equivalent comparative study with Experimental group (severe psychotic symptoms, low social  functioning) who received MCI-S mobile app + weekly mentoring sessions, vs Comparison group (light psychotic symptoms, good social functioning; received app only) | 10 weeks | 50 (24 severe symptom group, 26 relatively light symptom group) | SZ | - Change in personal and social functioning measured by the Personal and Social Performance Scale (PSP) and functions subscales – socially useful activities, personal and social relationships, self-care, disturbing and aggressive behaviors  - Change in maladaptive metacognitive beliefs measured by MCQ-30 and 5 subscales: Positive Beliefs About Worry, Negative Beliefs About Uncontrolled Ability and Danger of Worry, Cognitive Confidence, Need for Control, and Cognitive Self-Consciousness | - Total social function score significantly changed in the experimental group over time (p=0.001), but there was no significant change for the comparison group. No significant change in social functions subscales – socially useful activities, personal and social relationships, self-care, disturbing and aggressive behaviors.  - Meta-cognitive beliefs: Positive beliefs about worry shows significant changes between groups (p=0.016) and based on elapsed time (p=0.013) compared to the comparison group; Need for control was significantly different between groups (p=0.039); no statistically significant change in other scores. |
| Hanssen et al. *Psychiatry Res* 2020^16^ | SMARTapp | RCT of SMARTapp + personalized feedback vs SMARTapp, no personalized feedback | 21 days | 50 (Feedback 27, non-feedback 23) | SZ | - Social functioning: Social Functioning Scale (SFS) and subscales – social withdrawal, interpersonal functioning, prosocial activities, recreational activities | - No effect concluded of group on functioning (SFS) subscales – social withdrawal, interpersonal functioning, prosocial activities, recreational activities. |
| Kidd et al. *PLoS ONE* 2019^41^ | A4i | Interventional non-comparative study | 1 month | 38 allocated | SSD or other primary psychotic disorder | - Personal Recovery Outcome Measure (PROM) | - No significant improvements were seen in PROM after correcting for comparisons. |
| Lewis et al. *J Med Internet Res* 2020^17^ | ClinTouch | RCT of ClinTouch vs TAU | 12 weeks | 81 (intervention 40, control 41) | SZ and related disorders | - EuroQol 5D  - Global Assessment of Functioning scale (GAF) | - No significant difference in EuroQoL-5D scores between groups at 12 weeks (p>0.05) and no significant difference on GAF at Weeks 6 or 12. |
| Luther et al. *J Consult Clin Psychol* 2020^18^ | MEMS | RCT of MEMS + goal setting vs goal setting (control) | 8 weeks | 56 (intervention 27, control 29) | SSD | Quality of life: World Health Organization Quality of Life BRIEF  Functioning: Strauss-Carpenter Level of Function scale  - Effort-cost computations: EEfRT - Future reward-value representations: delay-discounting task  Overall goals attained | - No significant differences between groups in quality of life at the 8-week follow-up (p>0.05).  - No significant group differences for functioning (p>0.61).  - In an exploratory analyses, follow-up scores on the motivation item of the index were significantly greater in the MEMS group vs control after adjusting for baseline scores (p=0.04).  - No significant group effects for future reward-value representations (p=0.33), or effort-cost computations (p=0.70) (interpretation of ceiling effect and fixed responses).  - A significantly higher percentage of overall goals were attained with MEMS vs goal setting alone (p<0.001). |
| Moitra et al. *Psychiatric Quart* 2021^45^ | MACS | Interventional non-comparative study of MACS | 1 month | 10 | SSD | - Antipsychotic Medication Beliefs and Attitudes Scale (AMBAS) - World Health Organization Disability Assessment Schedule (WHODAS) | - No statistically significant improvements were reported for either AMBAS or WHODAS. |
| Röhricht et al. *BMC Psychiatry* 2021^19^ | Florence | RCT of Florence + TAU vs TAU | 6 months | 65 randomized (36 intervention, 29 control) | Severe mental illness (SZ, SZA, psychotic disorder unspecified, delusional disorder, BD) | - DIALOG scale (patient satisfaction scores on domain representing subjective quality of life measures) PROM and PREM  - General self-efficacy (GSE)  - Mental Health Confidence Scale (MHCS) | - No significant difference between intervention and control groups in patient satisfaction (DIALOG scores for PROM and PREM measures), GSE, or MHCS. |
| Schlosser et al. *Schizophr Bull* 2018^21^ | PRIME | RCT of PRIME vs TAU | 12 weeks | 43 (first allocated to PRIME, 22; first allocated to control, 21) | SSD | - Quality of life in social and vocational domains of QOL-A instrument - Real-world functioning in independent living, work, family, and social domains using the Role Functioning Scale (RFS)  - Self-efficacy: Revised Self-Efficacy Scale (R-SES)  - Motivated behavior: modified version of the Trust Task (reward learning)  - Dysfunctional Attitudes Scale (defeatist beliefs) | - No differences between groups in change from baseline of QOL-A or RFS scores at Week 12 (p>0.28). - Significant difference for self-efficacy on the R-SES (p=0.02); greater improvements from baseline to 12 weeks for PRIME vs wait list (WL) (p=0.02).  - While there was a trend towards significant improvement in learning from positive outcomes (reward learning), it was not significant (p=0.07).  - Significant difference for defeatist beliefs (p=0.02) vs TAU and vs WL (p=0.03). |
| Vitger et al. *J Med Internet Res* 2022^22^ | Digital shared decision-making smartphone app | RCT of smartphone app + TAU vs TAU alone (control) | 6 months | 194 (96 intervention, 98 control) | SZ, schizotypical or delusional disorder | - Data from Danish National Patient Register-Psychiatry: number of hospital admissions; length of admissions in days; adherence to OPUS appointments  - Global Assessment of Functioning (GAF)  - Personal and Social Performance Scale (PSP)  - General Self-Efficacy (GSE)  Consumer Health Activation Health Index – mental health version (CHAI-MH)  Perceived Efficacy in Patient-Physician Interactions (PEPPI)  Preparation for Decision-Making (PrepDM)  Working Alliance Inventory – Short (WAI-S)  Working Alliance Inventory – Short, provider version (WAI-S [P])  Service Engagement Scale (SES) | - No statistically significant differences between the groups for number of hospitalizations, length of admission or adherence to appointments.  - No statistically significant differences between the groups for GAF, PSP or GSE.  - Statistically significant improvements were seen in CHAI-MH (p=0.01), PEPPI (p=0.05) and PrepDM (p=0.04).  - No statistically significant differences between groups were seen for WAI-S (p=0.08) WAI-S (P) (p=0.34) or SES (p=0.60). |
| Wester-mann et al.  *J Consult Clin Psychol* 2020^24^ | iCBTp | RCT of iCBTp + TAU vs wait list (WL; control group with TAU for 8-wk RCT period, subsequently given access to iCBTp) | RCT: 8 weeks. Follow-up assessment (including wait-list control receiving intervention after 8 weeks): 6 months] | 101 (Intervention 50, control 51) | SSD | Psychological quality of life (WHOQOL; World Health Organization Quality of Life)  - Rosenberg Self-Esteem Scale (RSE)  - Mindful Attention and Awareness Scale (MAAS)  - Brief Interpersonal Competence Questionnaire (ICQ)  - Brief version of the incongruence questionnaire (K-INK)  - Internalized stigma of mental illness (ISMI) | - Significant interaction (p<0.05) for WHOQOL, RSE, ICQ, and psychological quality of life was seen in the PP but not ITT population and was not significant after correction for alpha error inflation.  - Significant interaction for MAAS among secondary outcomes with ITT analysis (no significant effect for other outcomes). - At the 6-month follow-up, the positive effects on all secondary outcomes (e.g., self-esteem) increased further or remained stable. During follow-up, no outcomes significantly deteriorated. |

#### Suppl. Table 9. Outcomes with no significant differences between groups (comparative studies) or between pre- and post-intervention (non-comparative studies) for Research Question 4: *What is the impact of DTs in schizophrenia/SSD on real-world functioning, quality of life, and other outcomes?*

| **Comparative studies** | | | | | | | | | | | |
| --- | --- | --- | --- | --- | --- | --- | --- | --- | --- | --- | --- |
| **DT** | **Control** | **Outcomes with no significance between-group difference (DT versus control)** | | | | | | | | | |
|  |  | **Functioning** | **QoL** | **HCRU** | **Stigma** | **Patient-provider engagement** | **Insight** | **Competency/**  **self-efficacy** | **Reward**  **responsivity** | **Medication**  **beliefs** | **Other outcomes** |
| SAVVy + TAU^1^ | TAU |  |  |  |  |  |  |  |  |  | ✓ ^a^ |
| FOCUS^3^ | TAU | ✓ ^a^ |  |  |  |  |  |  |  |  |  |
| T4RP ^4^ | TAU |  |  | ✓ (3) ^b^ |  | ✓ ^a^ |  |  |  |  |  |
| CBT2go^6^ | TAU |  |  |  |  |  |  |  |  |  | ✓ ^a^ |
| SlowMo + TAU^8^ | TAU |  |  |  |  |  |  |  |  |  | ✓(2) ^a^ |
| PEAR-004 + TAU^10^ | Digital sham + TAU |  | ✓ (4) ^a^ |  |  |  |  |  |  |  |  |
| MA-CBSST^11^ | Device contact only^c^ | ✓(2 [1^a^ + 1^b^]) |  |  |  |  | ✓ ^a^ |  |  |  | ✓ ^a^ (2 [1^a^ + 1^b^]) |
| EMPOWER^13^ | TAU | ✓ ^b^ |  |  |  | ✓ (5 [1^a^ + 4^d^]) | ✓ 4^a^ | ✓ ^a^ |  |  | ✓ ^b^ |
| MCI-S mobile app + weekly mentoring sessions^15^ | MCI-S mobile app | ✓ (4)^b^ |  |  |  |  | ✓(4 [3^a^ +1^b^]) |  |  |  |  |
| SMARTapp + personalized feedback^16^ | SMARTapp, no personalized feedback | ✓ (4) ^a^ |  |  |  |  |  |  |  |  |  |
| ClinTouch + TAU^17^ | TAU | ✓ ^b^ | ✓ ^a^ |  |  |  |  |  |  |  |  |
| MEMS + goal setting^18^ | Goal setting | ✓ (2) ^b^ | ✓ ^a^ |  |  |  |  |  | ✓ (2) ^b^ |  |  |
| Florence + TAU^19^ | TAU |  | ✓ (2) ^a^ |  |  |  |  | ✓ (2) ^a^ |  |  |  |
| PRIME ^21^ | WL + TAU | ✓ ^b^ | ✓ ^a^ |  |  |  |  |  | ✓ ^b^ |  |  |
| Smartphone app + TAU^22^ | TAU | ✓ (2) ^b^ |  | ✓ (3) ^b^ |  | ✓  (3 [1^a^ + 1^a/b/e^ + 1^d^]) |  | ✓ ^a^ |  |  |  |
| iCBTp + TAU^24^ | WL + TAU |  | ✓ ^a, f^ |  | ✓ ^a, f^ |  |  | ✓ (2) ^a, f^ |  |  | ✓ ^a^ |
| **Non-comparative studies** | | | | | | | | | | | |
| **DT** | | **Outcomes demonstrating no significant difference between pre-and post DT intervention** | | | | | | | | | |
|  |  | **Functioning** | **QoL** | **HCRU** | **Stigma** | **Patient-provider engagement** | **Insight** | **Competency/**  **self-efficacy** | **Reward**  **responsivity** | **Medication**  **beliefs** | **Other outcomes** |
| FOCUS^27^ | |  |  |  |  |  |  |  |  | ✓ (2) ^a^ |  |
| Self-management mobile app (unbranded)^29^ | | ✓ (6) ^a^ |  |  |  |  |  |  |  |  |  |
| weCOPE^31^ | |  | ✓ (2) ^a^ |  |  |  |  | ✓ ^a^ |  |  |  |
| MASS^35^ | | ✓ ^b^ |  |  |  |  |  |  |  |  |  |
| CBT2go^37^ | |  |  |  |  |  |  |  |  |  | ✓ ^a^ |
| MATS^38^ | | ✓ ^a^ |  |  |  |  |  |  |  |  |  |
| A4i^41^ | |  | ✓^a, g^ |  |  |  |  |  |  |  |  |
| MACS^45^ | | ✓ ^a^ |  |  |  |  |  |  |  | ✓ ^a^ |  |

^a^Self-reported; ^b^clinician/researcher/interviewer-administered assessment; ^c^device contact and symptom monitoring without CBSST; ^d^carer-assessed; ^e^based on patient records/data; ^f^intention to treat population; ^g^after correcting for multiple comparisons. Where there is more than 1 outcome type in a table cell, the number is given in brackets.

Outcomes with significant differences between groups or between pre- and post-intervention for Research Question 4 are shown in **Table 3**.

DT, digital therapeutic; HCRU, healthcare resource utilization; QoL, quality of life; TAU, treatment as usual. For full DT names, see Appendix at the end of this supplement.

# References

**1**. Bell IH, Rossell SL, Farhall J, Hayward M, Lim MH, Fielding-Smith SF, Thomas N. Pilot randomised controlled trial of a brief coping-focused intervention for hearing voices blended with smartphone-based ecological momentary assessment and intervention (SAVVy): feasibility, acceptability and preliminary clinical outcomes. *Schizophr Res* 2020;216:479-487.

**2.** Moore E, Williams A, Bell I, Thomas N. Client experiences of blending a coping-focused therapy for auditory verbal hallucinations with smartphone-based ecological momentary assessment and intervention. *Internet Interv* 2020;19:100299.

**3.** ClinicalTrials.gov. Available at: https://clinicaltrials.gov/study/NCT01969500. Accessed Jan 2025.

**4.** Cullen BA, Rodriguez K, Eaton WW, Mojtabai R, Von Mach T, Ybarra ML. Clinical outcomes from the texting for relapse prevention (T4RP) in schizophrenia and schizoaffective disorder study. *Psychiatry Res* 2020;292:113346.

**5.** Ybarra ML, Rodriguez KM, Fehmie DA, Mojtabai R, Cullen B. Acceptability of texting 4 relapse prevention, text messaging-based relapse prevention program for people with schizophrenia and schizoaffective disorder. *J Nerv Ment Dis* 2022;210(2):123-128.

**6.** Depp CA, Perivoliotis D, Holden J, Dorr J, Granholm EL. Single-session mobile-augmented intervention in serious mental illness: a three-arm randomized controlled trial. *Schizophr Bull* 2019;45(4):752-762.

**7.** Gallinat C, Moessner M, Apondo S, Thomann PA, Herpertz SC, Bauer S. Feasibility of an intervention delivered via mobile phone and internet to improve the continuity of care in schizophrenia: a randomized controlled pilot study. *Int J Environ Res Public Health* 2021;18(23):12391.

**8.** Garety P, Ward T, Emsley R, et al. Effects of slowMo, a blended digital therapy targeting reasoning, on paranoia among people with psychosis: a randomized clinical trial. *JAMA Psychiatry* 2021;78(7):714-725.

**9.** Greenwood KE, Gurnani M, Ward T, et al. The service user experience of slowMo therapy: a co-produced thematic analysis of service users' subjective experience. *Psychol Psychother* 2022;95(3):680-700.

**10.** Ghaemi SN, Sverdlov O, van Dam J, Campellone T, Gerwien R. A smartphone-based intervention as an adjunct to standard-of-care treatment for schizophrenia: randomized controlled trial. *JMIR Form Res* 2022;6(3):e29154.

**11.** Granholm E, Holden J, Dwyer K, Link P. Mobile-assisted cognitive-behavioral social skills training in older adults with schizophrenia. *J Behav Cog Ther* 2020;30(1):13-21.

**12.** Allan S, Beedie S, McLeod HJ, et al. Using EMPOWER in daily life: a qualitative investigation of implementation experiences. *BMC Psychiatry* 2023;23(1):597.

**13.** Gumley AI, Bradstreet S, Ainsworth J, et al. The EMPOWER blended digital intervention for relapse prevention in schizophrenia: a feasibility cluster randomised controlled trial in Scotland and Australia. *Lancet Psychiatry* 2022;9(6):477-486.

**14.** Gumley AI, Bradstreet S, Ainsworth J, et al. Digital smartphone intervention to recognise and manage early warning signs in schizophrenia to prevent relapse: the EMPOWER feasibility cluster RCT. *Health Technol Assess* 2022;26(27):1-174.

**15.** Han M, Lee K, Kim M, Heo Y, Choi H. Effects of a metacognitive smartphone intervention with weekly mentoring sessions for individuals with schizophrenia: a quasi-experimental study. *J Psychosoc Nurs Ment Health Serv* 2023;61(2):27-37.

**16.** Hanssen E, Balvert S, Oorschot M, Borkelmans K, van Os J, Delespaul P, Fett AK. An ecological momentary intervention incorporating personalised feedback to improve symptoms and social functioning in schizophrenia spectrum disorders. *Psychiatry Res* 2020;284:112695.

**17.** Lewis S, Ainsworth J, Sanders C, et al. Smartphone-enhanced symptom management in psychosis: open, randomized controlled trial. *J Med Internet Res* 2020;22(8):e17019.

**18.** Luther L, Fischer MW, Johnson-Kwochka AV, Minor KS, Holden R, Lapish CL, McCormick B, Salyers MP. Mobile enhancement of motivation in schizophrenia: a pilot randomized controlled trial of a personalized text message intervention for motivation deficits. *J Consult Clin Psychol* 2020;88(10):923-936.

**19.** Röhricht F, Padmanabhan R, Binfield P, Mavji D, Barlow S. Simple mobile technology health management tool for people with severe mental illness: a randomised controlled feasibility trial. *BMC Psychiatry* 2021;21(1):357.

**20.** Schlosser D, Campellone T, Kim D, Truong B, Vergani S, Ward C, Vinogradov S. Feasibility of PRIME: a cognitive neuroscience-informed mobile app intervention to enhance motivated behavior and improve quality of life in recent onset schizophrenia. *JMIR Res Protoc* 2016;5(2):e77.

**21.** Schlosser DA, Campellone TR, Truong B, Etter K, Vergani S, Komaiko K, Vinogradov S. Efficacy of PRIME, a mobile app intervention designed to improve motivation in young people with schizophrenia. *Schizophr Bull* 2018;44(5):1010-1020.

**22.** Vitger T, Hjorthøj C, Austin SF, Petersen L, Tønder ES, Nordentoft M, Korsbek L. A smartphone app to promote patient activation and support shared decision-making in people with a diagnosis of schizophrenia in outpatient treatment settings (momentum trial): randomized controlled assessor-blinded trial. *J Med Internet Res* 2022;24(10):e40292.

**23.** Ludtke T, Ruegg N, Moritz S, Berger T, Westermann S. Insight and the number of completed modules predict a reduction of positive symptoms in an Internet-based intervention for people with psychosis. *Psychiatry Res* 2021;306:114223.

**24.** Westermann S, Rüegg N, Lüdtke T, Moritz S, Berger T. Internet-based self-help for psychosis: findings from a randomized controlled trial. *J Consult Clin Psychol* 2020;88(10):937-950.

**25.** Achtyes ED, Ben-Zeev D, Luo Z, et al. Off-hours use of a smartphone intervention to extend support for individuals with schizophrenia spectrum disorders recently discharged from a psychiatric hospital. *Schizophr Res* 2019;206:200-208.

**26.** Ben-Zeev D, Scherer EA, Gottlieb JD, et al. mHealth for schizophrenia: patient engagement with a mobile phone intervention following hospital discharge. *JMIR Ment Health* 2016;3(3):e34.

**27.** Ben-Zeev D, Brenner CJ, Begale M, Duffecy J, Mohr DC, Mueser KT. Feasibility, acceptability, and preliminary efficacy of a smartphone intervention for schizophrenia. *Schizophr Bull* 2014;40(6):1244-1253.

**28.** Ben-Zeev D, Brian RM, Aschbrenner KA, Jonathan G, Steingard S. Video-based mobile health interventions for people with schizophrenia: Bringing the "pocket therapist" to life. *Psychiatr Rehabil J* 2018;41(1):39-45.

**29.** Cinemre B, Gulerce M, Gulkesen KH. A self-management app for patients with schizophrenia: a pilot study. *Stud Health Technol Inform* 2022;289:357-361.

**30.** de Almeida RS, Sousa TJ, Couto AS, Marques J, Queiros C, Martins CL. Development of weCope, a mobile app for illness self-management in schizophrenia. *Arch Clin Psychiatry* 2019;46(1).

**31.** de Almeida R, Couto A, Antonio Marques C, Queirs C, Martins C. Mobile application for self-management in schizophrenia: a pilot study. *J Tech Human Serv* 2018;36(4):179-190.

**32.** Eisner E, Bucci S, Berry N, Emsley R, Barrowclough C, Drake RJ. Feasibility of using a smartphone app to assess early signs, basic symptoms and psychotic symptoms over six months: a preliminary report. *Schizophr Res* 2019;208:105-113.

**33.** Eisner E, Drake RJ, Berry N, Barrowclough C, Emsley R, Machin M, Bucci S. Development and long-term acceptability of ExPRESS, a mobile phone app to monitor basic symptoms and early signs of psychosis relapse. *JMIR Mhealth Uhealth* 2019;7(3):e11568.

**34.** Fulford D, Mote J, Gard DE, Mueser KT, Gill K, Leung L, Dillaway K. Development of the motivation and skills support (MASS) social goal attainment smartphone app for (and with) people with schizophrenia. *J Behav Cogn Ther* 2020;30(1):23-32.

**35.** Fulford D, Gard DE, Mueser KT, Mote J, Gill K, Leung L, Mow J. Preliminary outcomes of an ecological momentary intervention for social functioning in schizophrenia: pre-post study of the motivation and skills support app. *JMIR Ment Health* 2021;8(6):e27475.

**36.** Fulford D, Schupbach E, Gard DE, Mueser KT, Mow J, Leung L. Do cognitive impairments limit treatment gains in a standalone digital intervention for psychosis? A test of the digital divide. *Schizophr Res Cogn* 2022;28:100244.

**37.** Granholm E, Holden J, Dwyer K, Mikhael T, Link P, Depp C. Mobile-assisted cognitive behavioral therapy for negative symptoms: open single-arm trial with schizophrenia patients. *JMIR Ment Health* 2020;7(12):e24406.

**38.** Granholm E, Ben-Zeev D, Link PC, Bradshaw KR, Holden JL. Mobile assessment and treatment for schizophrenia (MATS): a pilot trial of an interactive text-messaging intervention for medication adherence, socialization, and auditory hallucinations. *Schizophr Bull* 2012;38(3):414-425.

**39.** Grasa E, Seppälä J, Alonso-Solis A, et al. m-RESIST, a mobile herapeutic intervention for treatment-resistant schizophrenia: feasibility, acceptability, and usability study. *JMIR Form Res* 2023;7:e46179.

**40.** Huerta-Ramos E, Marcó-García S, Escobar-Villegas MS, et al. m-RESIST, a complete m-health solution for patients with treatmentresistant schizophrenia: a qualitative study of user needs and acceptability in the Barcelona metropolitan area. *Actas Esp Psiquiatr* 2017;45(6):277-289.

**41.** Kidd SA, Feldcamp L, Adler A, Kaleis L, Wang W, Vichnevetski K, McKenzie K, Voineskos A. Feasibility and outcomes of a multi-function mobile health approach for the schizophrenia spectrum: App4Independence (A4i). *PLoS One* 2019;14(7):e0219491.

**42.** Sequeira L, Kassam I, D'Arcey J, et al. Exploring contextual factors impacting the implementation of and engagement with a digital platform supporting psychosis recovery: a brief report. *Transl Behav Med* 2023;13(12):896-902.

**43.** Kim SW, Lee GY, Yu HY, et al. Development and feasibility of smartphone application for cognitive-behavioural case management of individuals with early psychosis. *Early Interv Psychiatry* 2018;12(6):1087-1093.

**44.** Kreyenbuhl J, Record EJ, Himelhoch S, Charlotte M, Palmer-Bacon J, Dixon LB, Medoff DR, Li L. Development and feasibility testing of a smartphone intervention to improve adherence to antipsychotic medications. *Clin Schizophr Relat Psychoses* 2019;12(4):152-167.

**45.** Moitra E, Park HS, Gaudiano BA. Development and initial testing of an mHealth transitions of care intervention for adults with schizophrenia-spectrum disorders immediately following a psychiatric hospitalization. *Psychiatr Q* 2021;92(1):259-272.

**46.** Terp M, Jørgensen R, Laursen BS, Mainz J, Bjørnes CD. A smartphone app to foster power in the everyday management of living with schizophrenia: qualitative analysis of young adults' perspectives. *JMIR Ment Health* 2018;5(4):e10157.

**47.** Vaidyanathan U, Snipes C, Lakhan SE, Dorner-Ciossek C, Campellone T. Can people with schizophrenia form a working alliance with a digital therapeutic app: A pilot study. American Psychiatric Association Annual Meeting, May 20-24, 2023, Poster Proceedings; poster session 13, abstract 37.

**48.** Snipes C, Dorner-Ciossek C, Hare BD, Besedina O, Campellone T, Petrova M, Lakhan SE, Pratap A. Establishment and Maintenance of a Digital Therapeutic Alliance in People Living With Negative Symptoms of Schizophrenia: Two Exploratory Single-Arm Studies. *JMIR Ment Health* Jan 27 2025;12:e64959.

**49.** von Malachowski A, Schlier B, Austin SF, et al. IMPACHS: feasibility and acceptability of an m-health solution integrated into routine clinical treatment for psychosis. *Schizophr Res* 2022;240:150-152.

#### Appendix: Abbreviations

AMBAS, Antipsychotic Medication Beliefs and Attitudes Scale; A-QLS, Abbreviated Quality of Life Scale; BARS, Brief Adherence Rating Scale; BCIS, Beck Cognitive Insight Scale; BCSS, Brief Core Schema Scale; BD, bipolar disorder; BDI, Beck Depression Inventory; BMQ, Brief Medication Questionnaire; BNA, Brief Neurocognitive Assessment; BPRS, Brief Psychiatric Rating scale-18-item; Brief COPE, Brief Coping Orientation to Problems Experienced; BUES, Boston University Empowerment Scale; CAINS-MAP, Clinical Assessment Interview for Negative Symptoms - Motivation and Pleasure; CAT, Client’s Assessment of Treatment Scale; CBSST, cognitive behavioral social skills training; CBT, cognitive behavioral therapy; CBTp, cognitive behavioral therapy for psychosis; CHAI-MH, Consumer Health Activation Index for Mental Health; CI, confidence interval; CMT, Comprehensive Module Test; CSQ-8, Client Satisfaction Questionnaire-8 item; d, day; DC, device contact; DPAS, Defeatist Performance Attitudes Scale; EMA, ecological momentary assessment; EMI, ecological momentary intervention; ESM, Experience Sampling Method; FoRSE, Fear of Recurrence Scale; GCT, generalized computer training; GPTS, Green et al. Paranoid Thoughts Scale; HADS, Hospital Anxiety and Depression Scale; IADLS, Instrumental Activities of Daily Living Scale; IBI, internet-based intervention; ILSS, Independent Living Skills Survey; ISI, Insomnia Severity Index; MA-CBSST, mobile-assisted cognitive-behavioral social skills training; MADRS, Montgomery-Asberg Depression Rating Scale; MAP-SR, Motivation and Pleasure Scale – Self-Report; MARS, Medication Adherence Rate Scale; MASC, Maryland Assessment of Social Competence; MATS, Mobile Assessment and Treatment of Schizophrenia; MEMS, Mobile Enhancement of Motivation in Schizophrenia; PANSS, Positive and Negative Syndrome Scale; PREM, Patient Reported Experience Measures (DIALOG); PROM, Patient Reported Outcome Measures (DIALOG); PSYRATS, Psychotic Symptom Rating Scales; QLS, Quality of Life Scale; QoL, quality of life; RAS-R, Recovery Assessment Scale Revised; RCT, randomized controlled trial; RFS, Role Functioning Scale; R-SES, Revised Self-Efficacy Scale; SAD, schizophrenia affective disorder; SANS, Scale for the Assessment of Negative Symptoms; SAPS, Scale for the Assessment of Positive Symptoms; SCT, social cognition training; SD, standard deviation; SDM, shared decision making; SFS, Social Functioning Scale; SMS, short message service; SSD, schizophrenia spectrum disorders; SZA, schizoaffective disorder; SZ, schizophrenia; SST: social skills training; TAM, Technology Acceptance Model; TAU, treatment as usual; WAI-S, Working Alliance Inventory - short form; Wks, weeks; WHODAS 2.0, World Health Organization Disability Assessment Schedule 2.0; WHOQOL, World Health Organization Quality of Life; WL, wait list; YMRS, Young Mania Rating Scale.
